# Quantitative detection and staging of presymptomatic cognitive decline in familial Alzheimer’s disease: a retrospective cohort analysis

**DOI:** 10.1186/s13195-020-00695-2

**Published:** 2020-10-06

**Authors:** Antoinette O’Connor, Philip S. J. Weston, Ivanna M. Pavisic, Natalie S. Ryan, Jessica D. Collins, Kirsty Lu, Sebastian J. Crutch, Daniel C. Alexander, Nick C. Fox, Neil P. Oxtoby

**Affiliations:** 1 Dementia Research Centre, UCL Queen Square Institute Of Neurology, 8-11 Queen Square, London, WC1N 3AR UK; 2 UK Dementia Research Institute at UCL, UCL, London, UK; 3 Department of Computer Science, UCL Centre for Medical Image Computing, 1st Floor, 90 High Holborn, London, WC1V 6LJ UK

**Keywords:** Preclinical, Neuropsychology, Familial Alzheimer’s disease, Event-based modelling

## Abstract

**Background:**

Understanding the earliest manifestations of Alzheimer’s disease (AD) is key to realising disease-modifying treatments. Advances in neuroimaging and fluid biomarkers have improved our ability to identify AD pathology in vivo. The critical next step is improved detection and staging of early cognitive change. We studied an asymptomatic familial Alzheimer’s disease (FAD) cohort to characterise preclinical cognitive change.

**Methods:**

Data included 35 asymptomatic participants at 50% risk of carrying a pathogenic FAD mutation. Participants completed a multi-domain neuropsychology battery. After accounting for sex, age and education, we used event-based modelling to estimate the sequence of cognitive decline in presymptomatic FAD, and uncertainty in the sequence. We assigned individuals to their most likely model stage of cumulative cognitive decline, given their data. Linear regression of estimated years to symptom onset against model stage was used to estimate the timing of preclinical cognitive decline.

**Results:**

Cognitive change in mutation carriers was first detected in measures of accelerated long-term forgetting, up to 10 years before estimated symptom onset. Measures of subjective cognitive decline also revealed early abnormalities. Our data-driven model demonstrated subtle cognitive impairment across multiple cognitive domains in clinically normal individuals on the AD continuum.

**Conclusions:**

Data-driven modelling of neuropsychological test scores has potential to differentiate cognitive decline from cognitive stability and to estimate a fine-grained sequence of decline across cognitive domains and functions, in the preclinical phase of Alzheimer’s disease. This can improve the design of future presymptomatic trials by informing enrichment strategies and guiding the selection of outcome measures.

## Background

Alzheimer’s disease (AD) is clinically characterised by slowly progressive cognitive decline that leads to multi-domain impairment and loss of independence. It is estimated that currently 50 million people worldwide are living with symptomatic AD with prevalence expected to triple by the year 2050 [1]. There is an urgent economic and social need for a treatment that can slow disease progression.

To date, clinical trials in symptomatic AD have failed to identify a disease modifying treatment. Possible reasons include inadequate enrichment/screening (non-responders will dilute any treatment effect) and insensitive cognitive end-points. In addition, it is increasingly acknowledged that there is a need for earlier intervention [2]. The pathological process of AD begins over a decade prior to symptom onset [3]. This clinically silent period opens up a treatment window at a potentially more tractable stage of the disease.

As we move towards presymptomatic trials, a greater understanding of the earliest cognitive changes of AD is needed. In particular, there is a need for sensitive measures of cognitive decline as future prevention trials will be assessed on cognitive end-points [4]. Although biomarkers will be important to detect disease effects ultimately, it will be critical to show benefit on cognitive and functional measures.

Familial Alzheimer’s disease (FAD) provides a unique opportunity to characterise the early changes in AD as this is a rare autosomal dominant condition where mutations are virtually 100% penetrant. FAD is caused by mutations in the Amyloid Precursor Protein (*APP*), Presenilin 1 (*PSEN1*), and Presenilin 2 (*PSEN2*) genes [5–7]. Despite its rarity, FAD displays similarities with typical AD in both clinical presentation and pathophysiology [8, 9].

It is now well recognised that presymptomatic cognitive changes occur in these kindreds. Deficits in verbal episodic memory have been most widely recognised, but impairments in processing speed, executive function and general intelligence have also been reported [10–12]. There is increasing interest in finding neuropsychological measures that can extend the interval over which presymptomatic cognitive decline is detectable; in particular, such measures would be very useful to complement biomarker changes. Measures of accelerated long-term forgetting (ALF), a process originally described in temporal lobe epilepsy, may be particularly sensitive to hippocampal dysfunction in AD [13, 14]. ALF refers to a process whereby new material is learned and retained normally, but is then forgotten at a more rapid rate than in those with normal hippocampal integrity and function. ALF has been shown to be a feature of asymptomatic *APOE*4 carriers and FAD mutation carriers at a time when performance on standard neuropsychological measures is unimpaired [14, 15].

In this study, we aimed to characterise the sequence and timing of cognitive decline in presymptomatic AD by analysing neuropsychological measures in an asymptomatic FAD cohort using an established data-driven computational model.

## Methods

### Study design and participants

We recruited participants from a longitudinal study of FAD at the Dementia Research Centre at University College London from January 2015 to April 2016. Participants are eligible for inclusion in this ongoing study if they are over 18 years of age and have a parent with genetically confirmed FAD or a clinical diagnosis of FAD. For this sub-study, participants needed to be asymptomatic, scoring zero on the Clinical Dementia Rating (CDR) scale [16], with neither they nor their informant reporting progressive cognitive decline. Participants were excluded from this sub-study if they had substantial co-existing neurological or psychiatric disease. The study was approved by the local Research Ethics Committee, and all participants provided written informed consent.

### Procedures

Genetic testing using Sanger sequencing was conducted to determine mutation status. Genetic results were provided only to statisticians to ensure that participants and study staff remained blind to genetic status. All participants identified an informant who was interviewed separately to gain a collateral history. Estimated years to symptom onset (EYO) was calculated by subtracting participants’ age at assessment from the age at which their affected parent first developed progressive cognitive decline using a semi-structured interview of family members; a negative value meaning that the participant was younger than the estimate of their probable age at onset.

Participants underwent a battery of neuropsychological assessments that cover a broad spectrum of cognitive domains and functions:

- General intelligence assessed using Performance IQ (PIQ) and Verbal IQ (VIQ) [17]. We also tested for a change in intellectual function using the dementia index, derived by subtracting the current IQ (measured by the Wechsler Abbreviated Scale of Intelligence) [17] from the predicted premorbid IQ (measured by the National Adult Reading Test) [18].
- Global cognition assessed using the Mini-Mental State Examination (MMSE) [19].
- Episodic memory:
  - Recognition Memory Test (RMT) words and faces [20].
  - Camden Paired Associate Learning (PAL) Test [21].
  - Accelerated long-term forgetting (ALF), using procedures reported previously [14]. The measures of interest were the proportion of material (list, story, figure) retained at 30 min that was later recalled at 7 days.
  - Subjective memory concerns were recorded using the Everyday Memory Questionnaire (EMQ) [22].
- Working memory assessed using digit span forwards and backwards.
- Timed executive function assessed using the Digit Symbol Substitution Test (DSST) [17].
- Inhibition assessed using the Stroop test [23].
- Arithmetic assessed using the Graded Difficulty Arithmetic (GDA) test [24].

### Statistical analysis

From cross-sectional data, we estimated the probable sequence of cognitive decline in presymptomatic FAD using an event-based model [25, 26]. The event-based model converts input data into severity scores (probability of abnormality) to estimate an order in which a set of measures become abnormal, and also estimate uncertainty in that ordering. The ordering and its uncertainty are inferred from the combinations of normal and abnormal measurements across individuals, using cross-sectional data, without defined cut-points, and without requiring any a priori staging variable (such as EYO). Due to the uniqueness of our cognitive battery, there exists no separate cohort for external validation, so the model was validated internally through cross-validation (described below).

We first removed healthy linear trends (if present, in noncarriers) of test score with years of formal education, age and sex. Event severity (probability of cognitive abnormality) in each test score is determined here using Kernel Density Estimation mixture modelling [25]. Using a mixture model replaces disease labels (mutation carrier/noncarrier) with pre-/post-event labels to allow for different cognitive profiles (and disease severity) across mutation carriers. This enables the sequence to be estimated without reference to an approximate staging variable (like EYO), which is not possible with standard groupwise comparisons. Data from a single visit per individual (the visit of their first ALF test) was used to fit the model.

The event-based model parameters are estimated using Markov Chain Monte Carlo (MCMC) sampling of the posterior distribution, which includes the maximum likelihood estimates and an explicit estimate of uncertainty [26].

The event-based model provides a fine-grained patient staging utility [27]. Based on their cognitive test score profile, participants were assigned to a numerical *model stage* reflecting their most probable position along the event sequence. This model stage reflects an individual’s current state of cumulative cognitive decline across the battery of tests.

Our model was cross-validated using repeated stratified 5-fold cross-validation (CV). This involves randomly splitting the cohort into 5 subset folds of approximately equal size and equal representation across groups (carriers/non-carriers). The event-based model is then fit (trained) on four folds and tested on the remaining fold, five times (using each fold as a test set once). This process was repeated for 50 different random 5-fold partitions, for a total of 250 trained models that are averaged to produce a robust and generalisable cross-validated model. We quantified similarity across CV folds using the Bhattacharyya coefficient which takes values between 0 and 1. We calculated accuracy of patient staging across folds using the final model to produce the ground truth model stage for all participants. We report details of cross-validation experiments in Additional file 1.

Presymptomatic trajectories of group-level cognitive decline were estimated from the cumulative time between cognitive decline events, estimated via Bayesian regression of EYO against model stage.

## Results

We first describe the demographics of our study participants, then present our experimental results on the sequence and timing of cognitive decline in presymptomatic FAD.

### Study participants

Participants’ demographic details are shown in Table 1. Of the 35 participants recruited, 21 were *APP* or *PSEN1* mutation carriers (Table e1, Additional file 1 for mutations) and 14 were non-carriers. The median EYO of mutation carriers was −6.5 years (interquartile range −9.2 to −5.0 years). Carriers and non-carriers were well-matched for age, sex EYO and years of education (see Table 1).

**Table 1:** Descriptive statistics and demographics of the FAD cohort in this study

	Mutation carriers	Non-carriers	***U*** (*χ^**2**^)	***p***
N	21	14	–	–
Age	38.9 (33.6, 42.0)	39.5 (33.9, 43.5)	131	0.30
Sex, M/F	11/10	6/8	*0.0429	0.84
EYO	− 6.5 (− 9.2, − 5.0)	− 7.7 (− 12.6, − 0.6)	134	0.34
Years of education	14 (12, 16)	14 (13, 17)	125	0.23
ALF– Story	66.8 (59.8, 81.9)	93.1 (85.1, 95.2)	47	0.0004
– Figure	70.2 (60.2, 79.4)	88.2 (77.7, 91.2)	45	0.0027
– List	43.7 (27.3, 59.7)	71.4 (58.8, 76.4)	54	0.0009
EMQ	18.3 (11.9, 27.6)	11.2 (8.7, 13.3)	62	0.0022
DSST	57.1 (47.1, 62.4)	69.1 (58.6, 74.5)	62	0.032
Digit Span – fwd	10.1 (7.4, 10.7)	10.0 (9.4, 11.6)	84	0.085
– back	8.9 (6.6, 9.6)	9.0 (6.9, 9.9)	110	0.31
PIQ	108.4 (98.0, 113.6)	116.9 (109.0, 126.4)	60	0.01
VIQ	101.8 (98.0, 107.4)	104.1 (97.3, 114.1)	100	0.26
Stroop	50.0 (45.0, 57.6)	47.8 (38.5, 48.7)	77	0.076
RMT– words	49.3 (48.6, 49.7)	49.0 (48.1, 49.3)	78	0.054
– faces	43.2 (40.7, 47.9)	43.5 (42.0, 45.6)	100	0.26
MMSE	29.7 (29.1, 29.9)	29.4 (29.3, 29.7)	84	0.14
Dementia Index	− 4.6 (− 7.9, 2.6)	− 3.7 (− 11.1, 2.6)	100	0.27
GDA	14.1 (8.8, 16.6)	16.7 (13.0, 19.5)	82	0.074
PAL	18.4 (14.2, 22.9)	18.4 (16.7, 19.9)	110	0.43

Features were adjusted for healthy linear trends in age, sex and gender (in non-carriers). Values quoted as median (interquartile range). Group comparisons were calculated using the Mann-Whitney *U* test (*χ*^**2**^ contingency test for sex)

*Abbreviations: EYO* estimated years to symptom onset, *M/F* male/female, *EMQ* Everyday Memory Questionnaire, *DSST* Digit Symbol Substitution Test, *PIQ/VIQ* Performance/Verbal Intelligence Quotient, *RMT* Recognition Memory Test, *MMSE* Mini-Mental State Examination, *GDA* Graded Difficulty Arithmetic, *PAL* Paired Associate Learning

### Sequence of presymptomatic cognitive decline

**Fig. 1:**
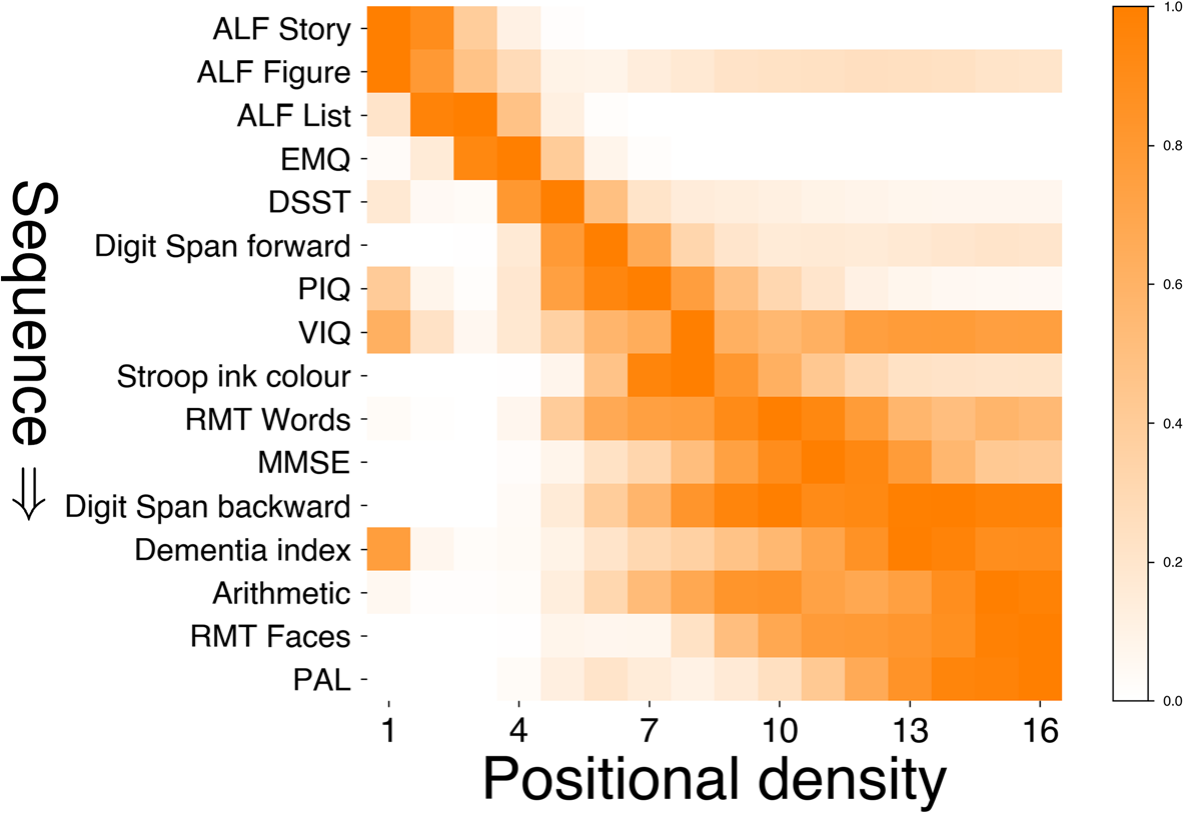
Estimated sequence of presymptomatic cognitive decline in FAD. Probabilistic heat map shows higher confidence (left-to-right) in the ordering (top-to-bottom) as hotter, narrow diagonal regions. Cooler, broader regions show lower confidence in the ordering of events. For abbreviations, see Table 1 caption

Figure 1 shows the data-driven sequence of cognitive decline events in presymptomatic FAD, and uncertainty in the sequence, represented as a positional density map. High confidence (left-to-right) in the ordering (top-to-bottom) appears as a narrow diagonal. Lower confidence appears as broader regions.

The model suggests the following sequence of cognitive decline in FAD: the first measure to become “abnormal” was ALF (story, figure and list), followed by subjective cognitive decline (EMQ), timed executive function (DSST), working memory (digit span: forwards), general intelligence (PIQ, subsequently VIQ) and inhibition (Stroop). Overlapping changes are then detected in other measures of episodic memory (RMT and later PAL), global cognition (MMSE) and arithmetic (GDA). This cross-validated model (see Statistical Methods and Additional file 1) reveals a robust and consistent sequence of presymptomatic cognitive decline: qualitatively, the positional density map is concentrated towards the diagonal, especially early in the sequence, and quantitatively, the Bhattacharyya coefficient for cross-validation similarity was 0.67.

**Fig. 2:**
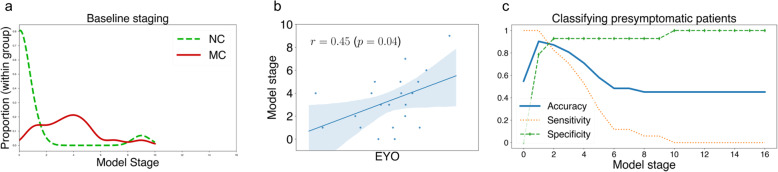
Model staging results. **a** Model stage distribution by mutation status. Smoothed histograms of individual model stage in mutation carriers (red) and noncarriers (broken green). Mutation carriers are at more-advanced stages, with all noncarriers except for one outlier being at stage one (ALF Story) or zero. This outlier demonstrated inconsistencies in cognitive performance with discordant scoring across tests of the same cognitive domain, e.g. low event probability for ALF list vs high event probability for ALF story. **b** Model stage correlates with EYO in mutation carriers. Model stage correlates with EYO (*x*-axis labels omitted to avoid unblinding). Linear regression gives *r*^2^ = 0.24 (*p* < 0.05). **c** Model stage can be used to classify patients (mutation carrier) from controls (noncarriers) at a very early presymptomatic stage, with very high accuracy (> 90%). Abbreviations: NC, noncarrier; MC, mutation carrier

Figure 2 demonstrates the staging potential of the model for this presymptomatic cohort. Each participant in the dataset was assigned a model stage that best reflects their measurements (see the “Methods” section and Young et al. [27]). Staging proportions for mutation carriers and noncarriers are shown in Fig. 2a, where a clear separation is seen between mutation carriers (advanced stages) and noncarriers (earliest stages, except for one outlier with a discordant cognitive profile; see Fig. 2 caption). This separation can be exploited to classify future patients (mutation carriers) from non-carriers in this presymptomatic cohort with very high accuracy, as shown in Fig. 2c—90% balanced accuracy is achieved by classifying participants at stage 2 (ALF Story + ALF Figure) or above as future patients. This suggests that our generative model may be useful for discriminative applications in preclinical Alzheimer’s disease. Figure 2b shows that model stage correlates with EYO in mutation carriers, despite EYO being completely separate from the model.

### Timing of presymptomatic cognitive decline

**Fig. 3:**
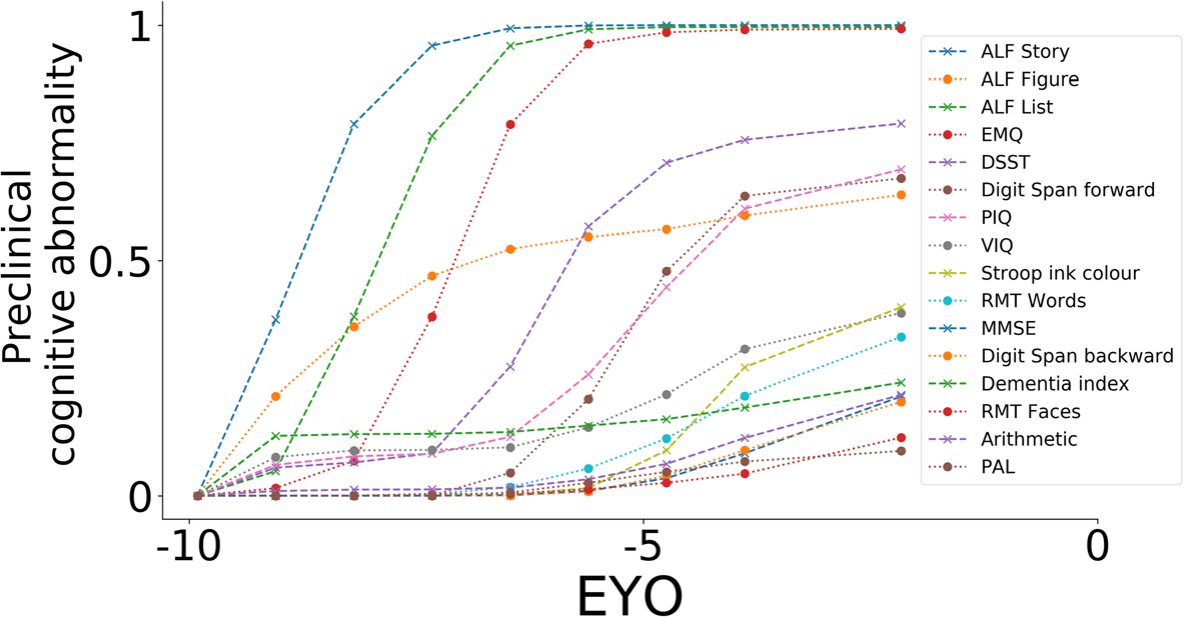
Data-driven preclinical cognitive decline in familial Alzheimer’s disease. Cumulative probability of preclinical abnormality as a function of years prior to estimated disease onset. A plateau means the data suggest that preclinical cognitive decline has stalled—it does not mean that the test score is at floor/ceiling. Progression sequence is that of the cross-validated event-based model in Fig. 1

Figure 3 shows our model-estimated curves for the timing of preclinical cognitive decline in this FAD cohort. Our model demonstrates data-driven cognitive abnormality as a function of EYO, estimated by combining our cross-validated model (Fig. 1) with EYO—see Statistical Methods and Additional file 1 for details. Our model first detects decline in measures of accelerated long-term forgetting; verbal measures (ALF Story and List) begin to change before visual measures (ALF Figure). In this asymptomatic cohort, our model estimates that changes in measures of ALF start approximately a decade prior to estimated symptom onset. Levels of self-reported subjective cognitive impairment also begin to increase around this time. Later changes are seen in measures of timed executive function (DSST) and general intelligence (PIQ). As estimated symptom onset approaches, subtle changes start to occur in measures of inhibition (Stroop), verbal intelligence (VIQ), global cognition (MMSE), memory (RMT, PAL, digit span), and arithmetic (GDA).

## Discussion

Our work uses data-driven modelling to assess the sequence, timing and uncertainties of presymptomatic cognitive decline, advancing existing understanding of cognitive impairment in preclinical AD and providing a mechanism for detailed staging of presymptomatic individuals. The probabilistic cascade broadly agrees with current knowledge of cognitive decline in Alzheimer’s disease. Here we discuss our results in the context of previous work and highlight new avenues for further investigation.

Previous studies in sporadic AD and FAD had demonstrated impairments in long-term retention (i.e. accelerated forgetting) in clinically normal individuals and have shown that severity correlates with proximity to symptom onset [14, 15]. Our data-driven modelling of a clinically normal FAD cohort provides an insight into the timing of presymptomatic cognitive decline. In this cohort, we found changes in measures of accelerated forgetting starting approximately a decade prior to estimated symptom onset. This timing overlaps with the onset of metabolic changes in fluorodeoxyglucose PET in previous studies of FAD [3]. Cerebral hypometabolism reflects synaptic dysfunction which would be expected to result in cognitive decline. However, cognitive changes this far from symptom onset have not been routinely detected by traditional neuropsychology measures.

Our results reveal a distinct ordering of cognitive decline in presymptomatic mutation carriers. Our model demonstrates early changes in tests of timed executive function, inhibition and performance IQ. This result is consistent with previous findings of early deficits in fronto-subcortical and general intellectual function in AD [10, 11, 28]. Preclinical change in measures of processing speed and executive function has also been reported in cognitively normal amyloid-positive individuals [29]. These results suggest that neuronal damage due to AD may be widespread even at an asymptomatic stage, highlighting the importance of conducting multi-domain cognitive testing in future presymptomatic studies.

Studies modelling cognition in preclinical late onset AD (LOAD) have also shown the value of multi-domain testing [30]. Cognitive composites, like the preclinical Alzheimer’s cognitive composite (PACC) which is a combined measure of episodic memory, executive function and global cognition, have demonstrated declining performance amongst asymptomatic amyloid-positive groups [31, 32]. We also found change in these domains amongst cognitively normal individuals on the FAD continuum. Additionally, our staging model demonstrates differences in the ordering of change across these individual domains, declines in sensitive measures of memory (ALF) preceding declines in executive function (DSST) and global cognition (MMSE). Similarly, individual measures within the PACC do not change uniformly [33]. Consistent with our findings in FAD, sensitive memory tests detect early decline in preclinical LOAD, potentially even before the threshold of amyloid positivity is reached, while measures of global cognition begin to change closer to symptom onset [33, 34].

Our results on the timing of presymptomatic cognitive decline reveal differences in verbal and visual measures. Abnormality in verbal measures of accelerated forgetting preceded changes in the visual measure; this needs to be replicated in a larger longitudinal study but implies verbal tests may be particularly adept at detecting early cognitive decline in FAD. This finding holds also with other verbal measures of episodic memory (RMT Words), which showed decline before visual measures (RMT Faces) in our study, as found previously [10]. Given that verbal episodic memory is critically dependent on the left hippocampus, whereas visual memory is more dependent on right hemispheric function, our results are consistent with a previous report of earlier left sided hippocampal atrophy in FAD [35]. However, this result needs to be interpreted with caution as there is no clear consensus on the hemispheric lateralisation of preclinical hippocampal atrophy in FAD [36].

There is growing interest in the clinical utility of subjective cognitive decline in AD. Memory concerns have demonstrated associations with pathological hallmarks of disease (cortical tau deposition as well as amyloid accumulation) [37, 38]. Subjective cognitive decline may, at least in some individuals, predict subsequent cognitive decline and progression to dementia. However, in FAD, there have been conflicting reports on the utility of measures of subjective cognitive decline [39, 40]. In our study, self-reported decline appeared early in the sequence of preclinical cognitive change. Our finding is consistent with a previous report of higher self-reported memory concerns in mutation carriers compared to non-carriers in an asymptomatic *PSEN1* kindred [40]. Similar results for predicting symptomatic progression in sporadic AD [41] add credence to the idea that some level of self-insight into cognitive difficulties is present during the preclinical course of AD.

Our finding that tests of different cognitive measures are sensitive to detecting change over different time periods is analogous to hypothetical models of AD progression [42] where biomarkers are dynamic during finite, sequential intervals. Here we only investigated the preclinical phase, so while the plateaus in Fig. 3 may suggest that preclinical cognitive decline has slowed, longitudinal follow-up into the symptomatic phase is needed to clarify the full dynamic range of these measures. Such longitudinal work will also be important to fully characterise cognitive trajectories, as non-uniform patterns of change across neuropsychology measures have previously been demonstrated in prodromal AD [43]. Notwithstanding this, our results may be helpful to inform the selection of neuropsychology measures in clinical testing, as well as the choice of screening and outcome measures in future trials. For example, in AD prevention trials, accelerated forgetting (ALF Story and List) and subjective cognitive decline (EMQ) may be useful for early screening, while other measures (e.g. Performance IQ) may be more useful as outcome measures. This could be operationalised using our model staging utility: individuals at earlier model stages might be deemed suitable for screening into an early intervention clinical trial, and model stage could be used longitudinally as an outcome measure. Model stage could also inform clinical decision making and prognostication; individuals at later model stage being at higher risk of subsequent progression.

Our model demonstrates a high degree of certainty for the staging of early events; however, there is significant overlap for later cognitive measures. Phenotypic heterogeneity, which will result in inter-individual variability in cognitive profiles [44], may be contributing to this uncertainty. Alternatively, overlay in event ordering may be due to these neuropsychology measures declining simultaneously. However, one needs to be cautious when interpreting the significance of our model staging, particularly for late events, as mutation carriers in this study were on average over 7 years away from estimated symptom onset. Individuals this many years from symptom onset often show little to no change in many neuropsychological measures [3, 11, 12]. Future work in larger FAD cohorts with greater sampling of the late preclinical phase is needed to better clarify the timing of cognitive changes across the preclinical-clinical watershed, which could include data-driven subtyping [45].

### Limitations

Our study has limitations. First, the sample size is small (*n* = 35). Therefore, replication of our findings in other, preferably large FAD cohorts, like the Dominantly Inherited Alzheimer’s Network, is desirable [46]. Ideally such a cohort would be followed into the symptomatic phase. Such longitudinal data could be used to test the longitudinal consistency of our model while also offering the potential to personalise the model using within-person cognitive change. Follow-up data also enables fitting of alternative disease progression models such as differential equation models which could also be used to inform cognitive trajectories and timings [47, 48]. Secondly, the known inaccuracies in EYO [49] limits the accuracy of our estimates of the timing of presymptomatic cognitive decline. Ideally, we would repeat our analysis on this same data using actual age of onset in place of EYO, after following all individuals through to symptom onset. Third, neuropsychological tests of any given domain are not uniformly sensitive, and inclusion of more sensitive measures may lead to changes in the position of that domain within the sequence. Finally, we did not account for practice effects, which tend to confound assessment of cognitive decline. This is also a strength of our study in that we were able to estimate the sequence and timing of presymptomatic cognitive decline, despite not removing this potential confounder.

## Conclusion

In summary, our study has exploited an established computational modelling approach to reveal novel insight into the precise nature of presymptomatic cognitive decline in AD, using only cross-sectional data from a unique battery of neuropsychological tests. We have argued in favour of the utility of our data-driven findings for enabling and empowering presymptomatic clinical trials in Alzheimer’s disease.

## Supplementary information

**Additional file 1.** Details of cross-validation experiments

## Data Availability

The datasets used and/or analysed during the current study are available from the corresponding author on reasonable request.

## Abbreviations

AD: Alzheimer’s disease
ALF: Accelerated long-term forgetting
*APP*: Amyloid Precursor Protein
CDR: Clinical Dementia Rating Scale
CV: Cross-validation
DSST: Digit Symbol Substitution Test
EMQ: Everyday Memory Questionnaire
EYO: Estimated years to symptom onset
FAD: Familial Alzheimer’s disease
GDA: Graded Difficulty Arithmetic
LOAD: late onset Alzheimer’s disease
MCMC: Markov Chain Monte Carlo
MMSE: Mini-Mental State Examination
PAL: Paired Associate Learning
PIQ: Performance IQ
*PSEN1*: Presenilin 1
*PSEN2*: Presenilin 2
RMT: Recognition Memory Test
VIQ: Verbal IQ
