# Supplementary material for: Quantitative detection and staging of presymptomatic cognitive decline in familial Alzheimer’s disease: a retrospective cohort analysis
Source: Alzheimers Res Ther. 2020 Oct 6;12:126. doi: 10.1186/s13195-020-00695-2 (PMC7539456; doi:10.1186/s13195-020-00695-2)
Supplement: Supplementary file 1 — Additional file 1. Details of cross-validation experiments [file 13195_2020_695_MOESM1_ESM.docx]

Supplementary Material:

Quantitative detection and staging of presymptomatic cognitive decline in familial Alzheimer's disease

Antoinette O’Connor, Philip S.J. Weston, Ivanna M. Pavisic, Natalie S. Ryan, Kirsty Lu, Sebastian J. Crutch, Daniel C. Alexander, Nick C. Fox, Neil P. Oxtoby

Here we provide supplementary information and results, including: the mixture modelling for quantifying event severity in each cognitive test score; detailed cross-validation results; method for estimating the timing of cognitive decline from the estimated sequence; and cross-sectional trends of cognitive test scores with estimated years to onset (EYO).

# 1. Methods: event severity

Within the event-based model (EBM), the probability of an event is generated in a data-driven manner by using a mixture model that allows patients (here mutation carriers) to be probabilistically labelled as pre-event or post-event. Doing this in such a data-driven manner is what enables the EBM to infer the sequence of cognitive decline using only cross-sectional data and without reliance upon EYO.

Supplementary Figure S1 shows the event severity measures and corresponding probability density functions (PDFs) for pre-event and post-event for each of the cognitive tests included in our model. We do not include the histograms to avoid unblinding of participants to their mutation status.

| 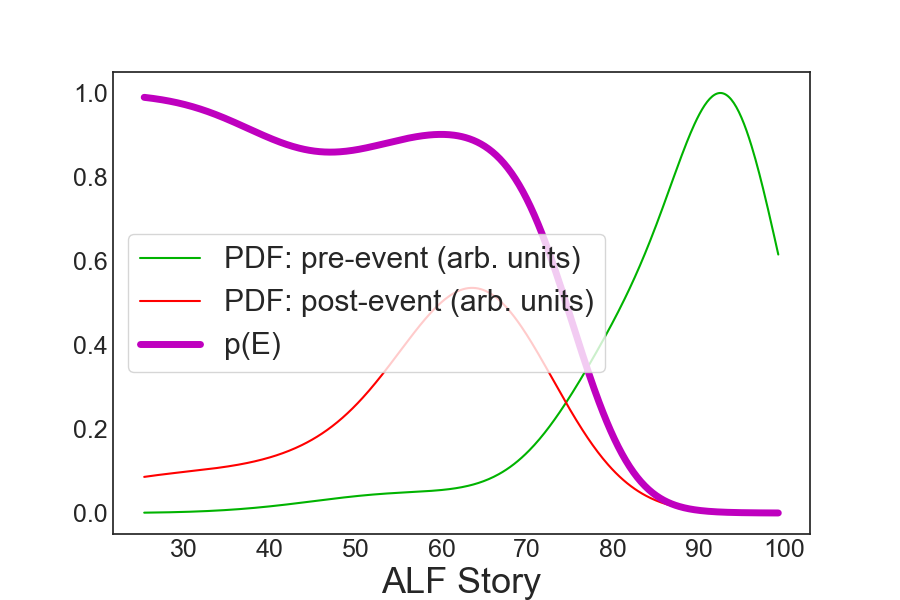 | 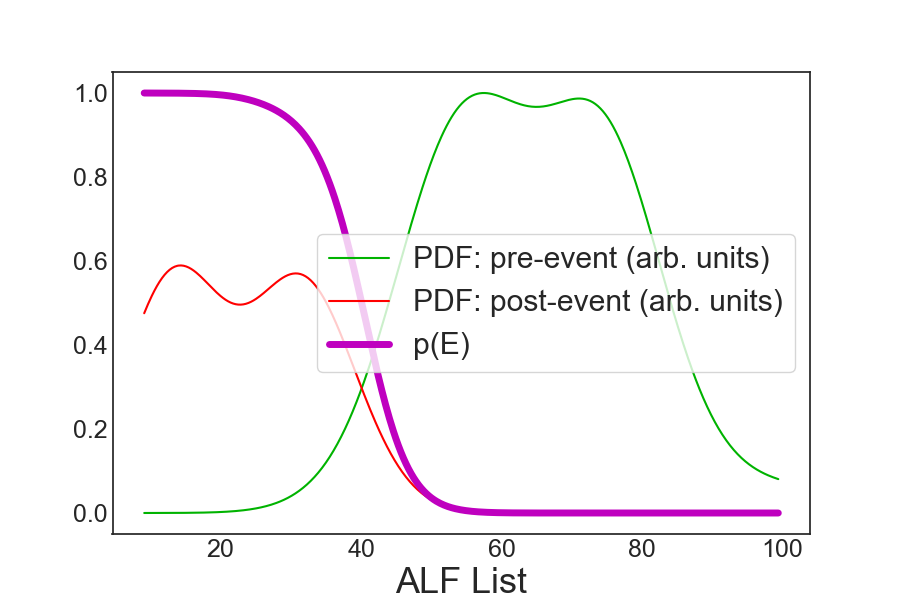 | 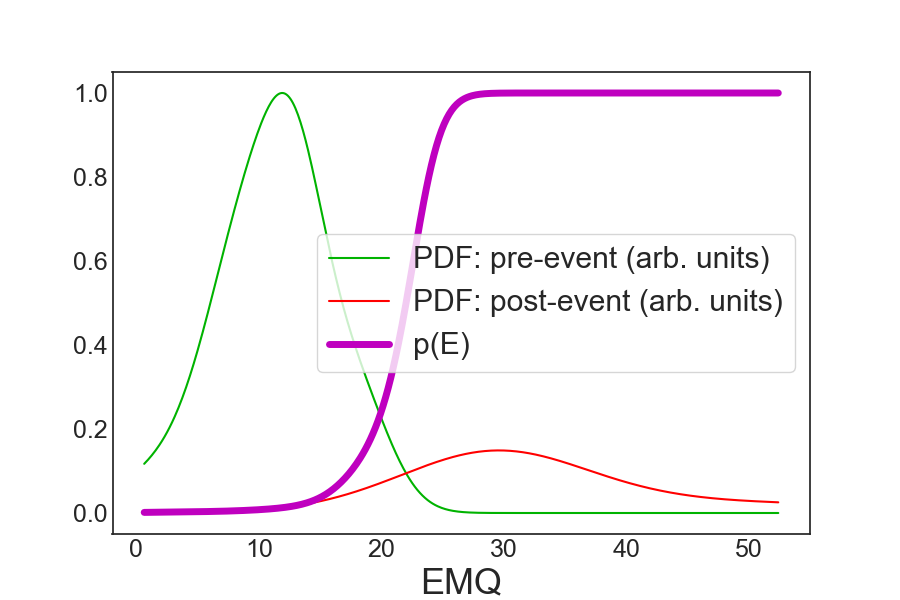 |
| --- | --- | --- |
| ALF Story | ALF List | Everyday Memory Questionnaire |
| 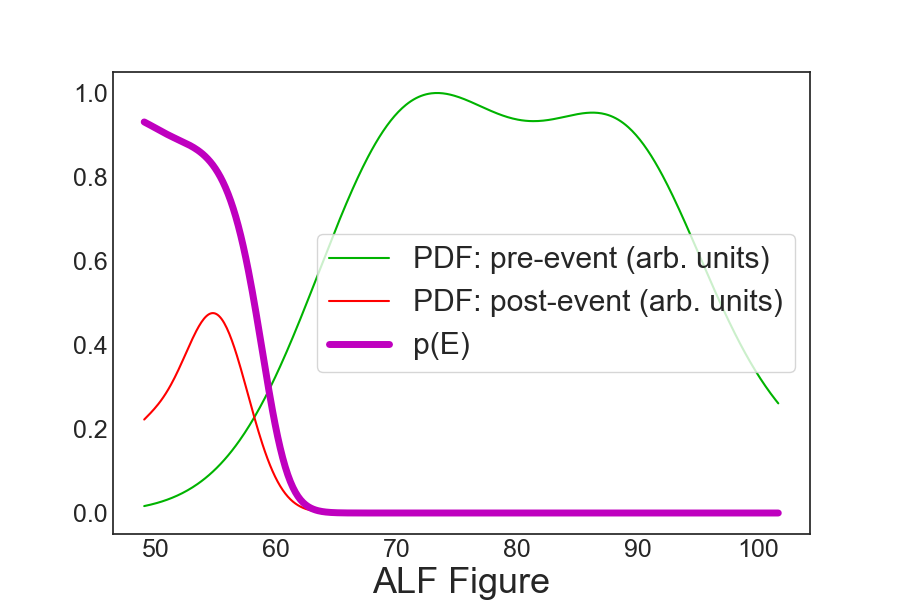 | 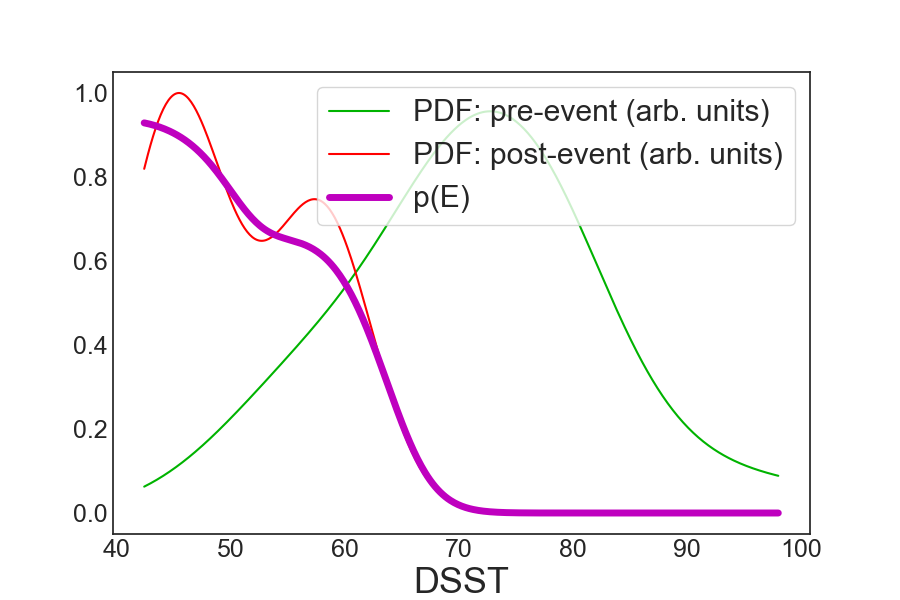 | 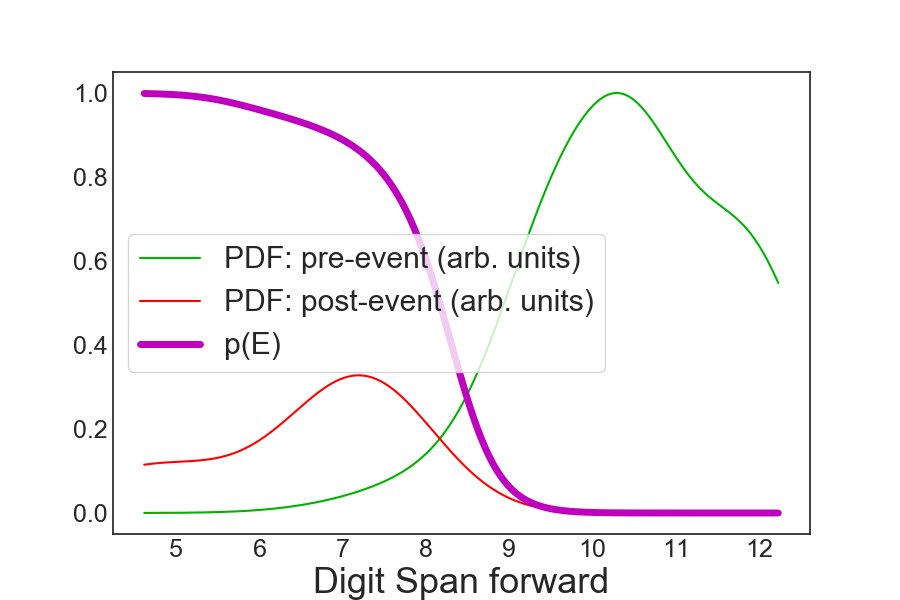 |
| ALF Figure | Digit Symbol Substitution | Digit Span forward |
| 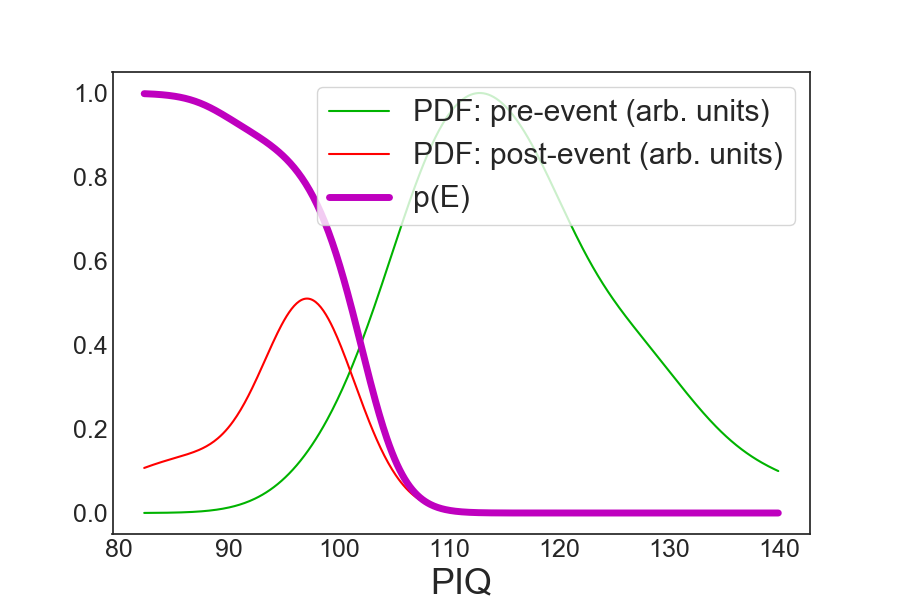 | 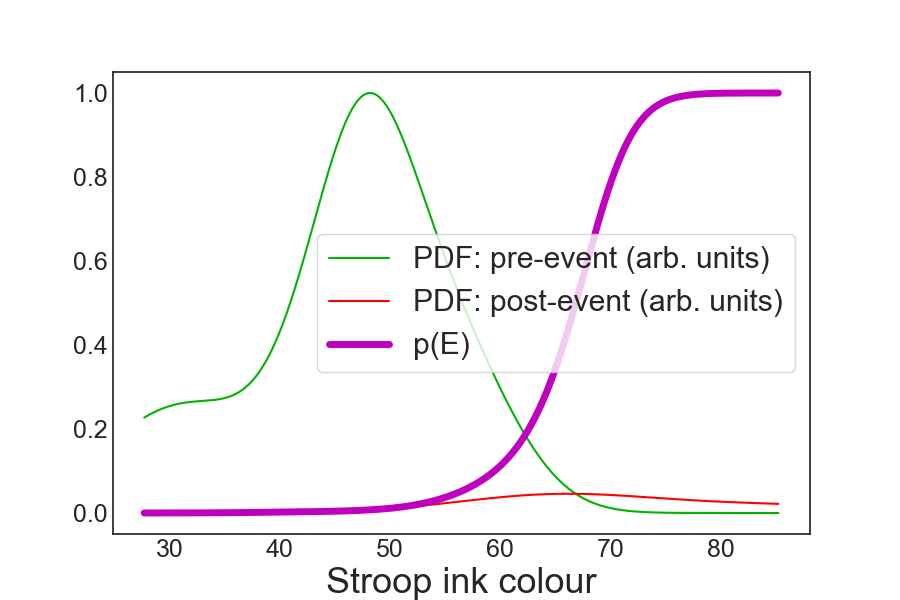 | 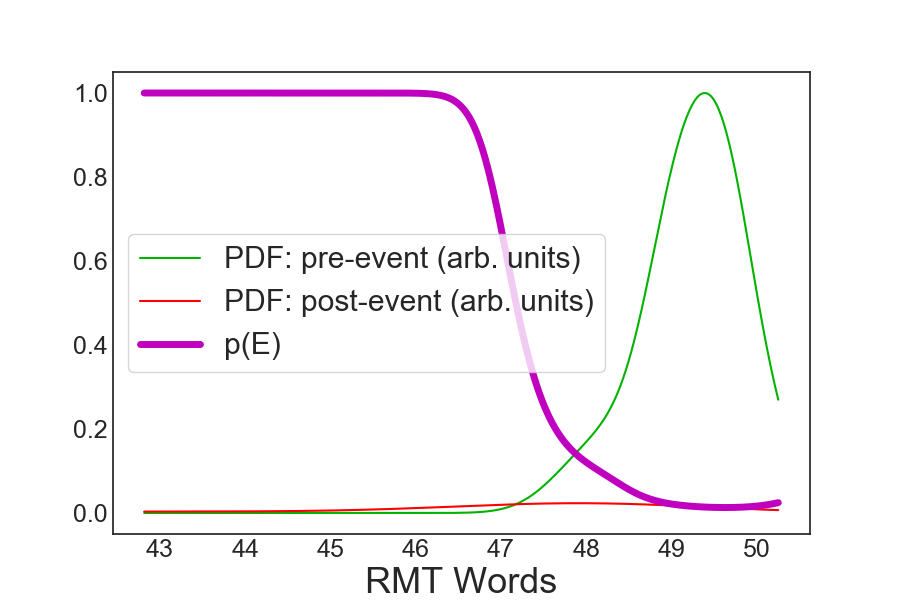 |
| Performance IQ | Stroop ink colour | Recognition Memory: Words |
| 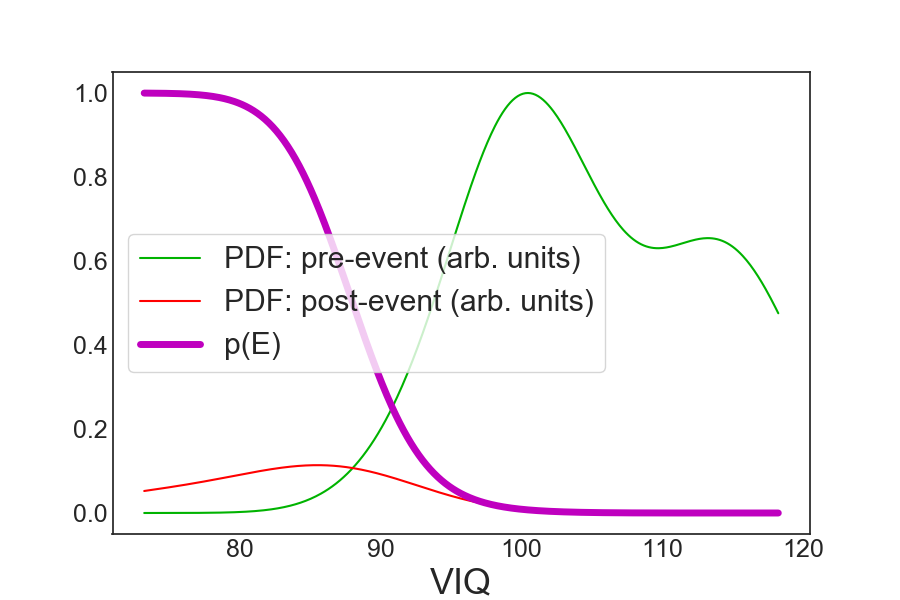 | 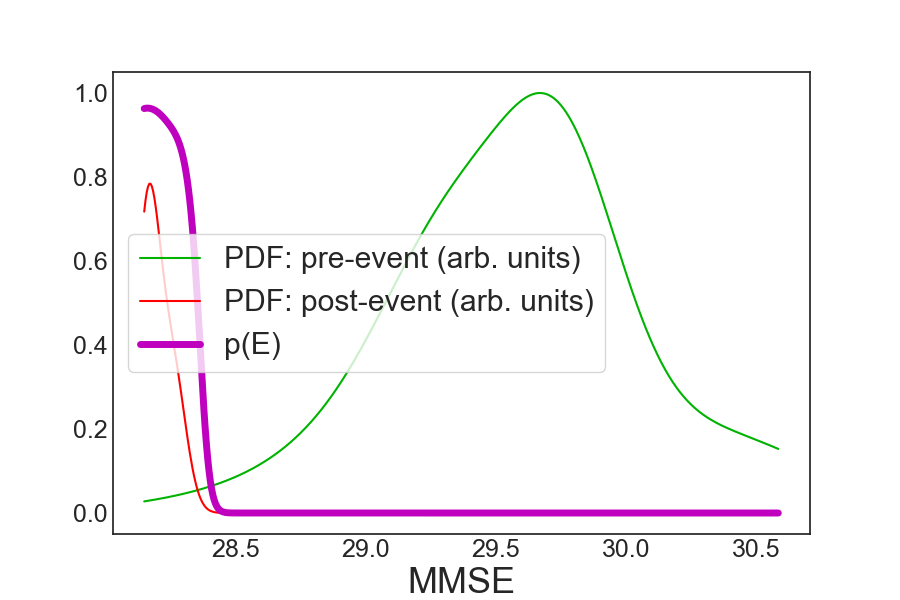 | 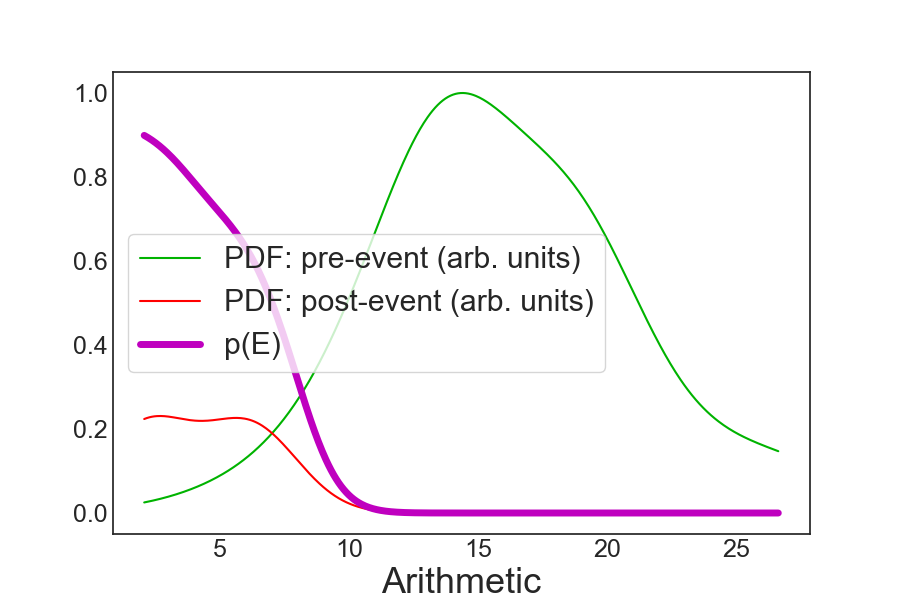 |
| Verbal IQ | MMSE | Graded Difficulty Arithmetic |
| 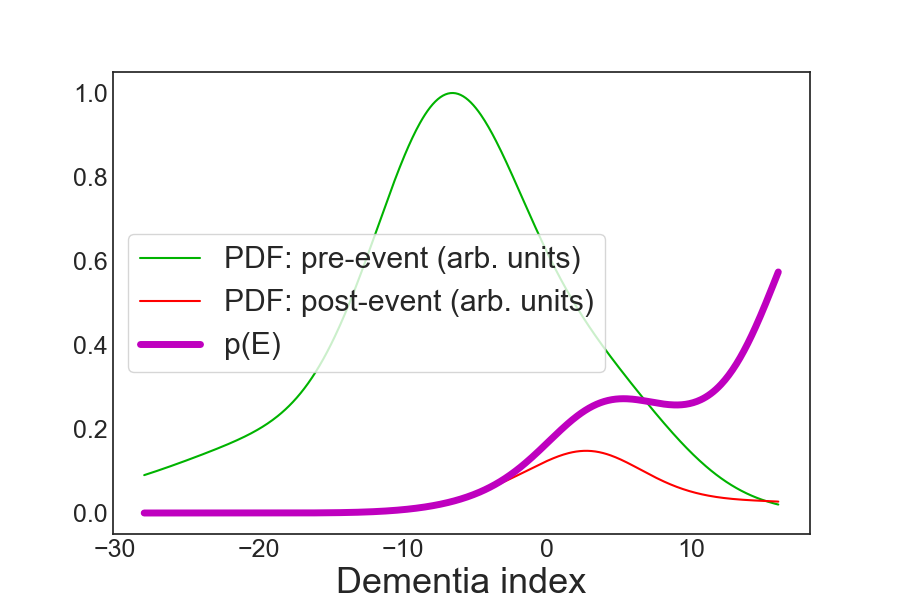 | 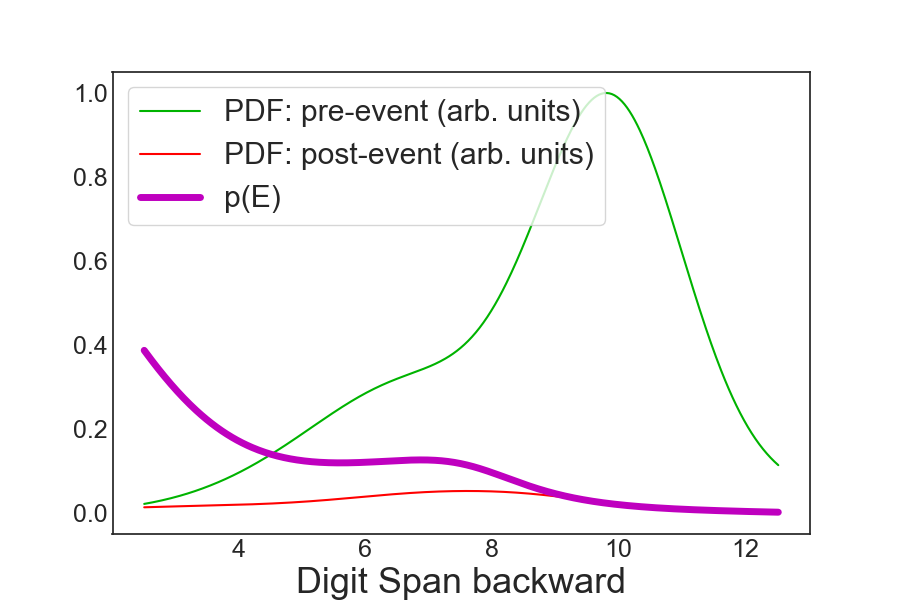 | 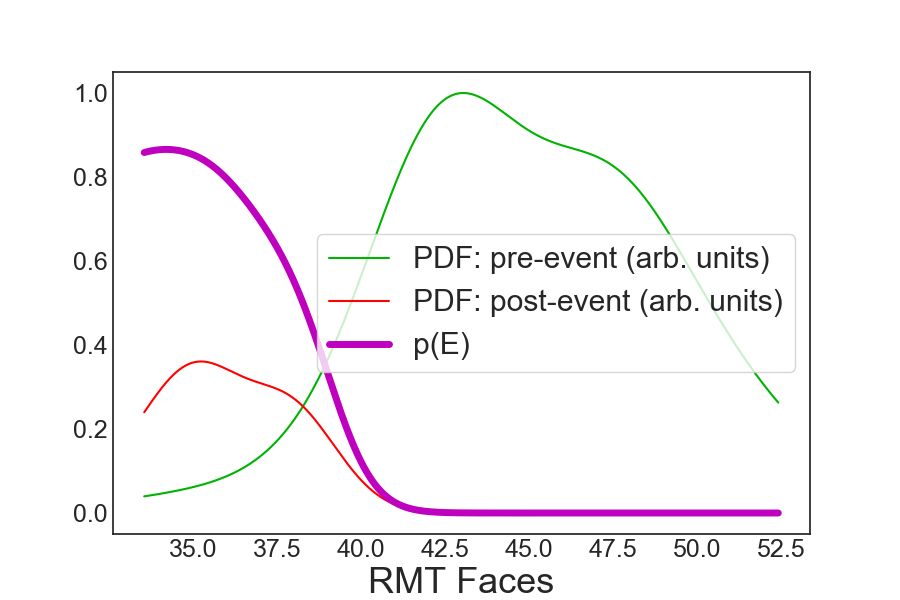 |
| Dementia index | Digit Span backward | Recognition Memory: Faces |
| 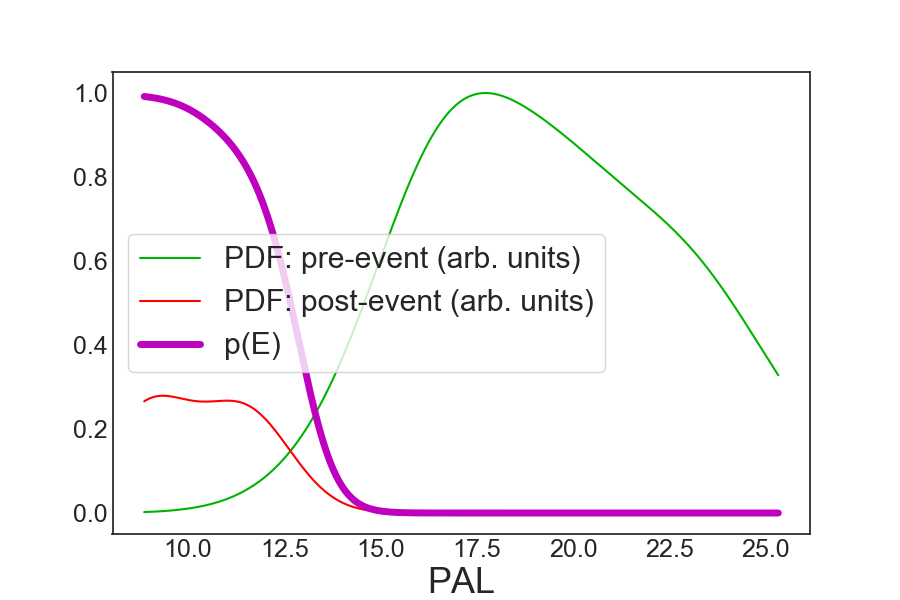 | **Figure S1**. Event severity for cognitive test scores. Pre-event distributions (green) are representative of noncarriers and relatively high-performing presymptomatic mutation carriers. Post-event distributions (red) correspond to relatively poorly-performing presymptomatic mutation carriers. Magenta curves showing the probability of an event as a function of test score. The event-based model combines event severity across participants to estimate the sequence of presymptomatic cognitive decline. Abbreviations: PDF – Probability Density Function; ALF – Accelerated Longitudinal Forgetting; EMQ – Everyday Memory Questionnaire; DSST – Digit Symbol Substitution Test; PIQ – Performance Intelligence Quotient (IQ); VIQ – Verbal IQ; RMT — Recognition Memory Test; MMSE – Mini Mental State Examination; PAL – Paired Associate Learning. | |
| Paired Associate Learning |  |  |

# 2. Cross-Validation

## We performed repeated stratified 5-fold cross-validation. The cohort is randomly split into 5 subset “folds” each having approximately the same size and approximately equal ratios of mutation carriers and non-carriers. In turn, each ~20% fold is held aside as a test set and the event-based model is built using the remaining ~80% of the data (training set). This 5-fold cross-validation procedure was repeated 50 times using different random partitions. Accuracy was assessed *post hoc* using patient staging: test subjects were assigned a model stage (see Methods section and [Young-2014]) within the corresponding trained model, which was compared with ground truth model stage estimated from the full cross-validated model.

## 2.1 Model consistency: cross-validation similarity

## Model consistency is assessed by quantifying statistical overlap of the posterior positional density across folds. To obtain a measure of similarity where 0 corresponds to no overlap, and 1 corresponds to identical distributions, we use the Bhattacharyya coefficient (1). Our 250 cross-validation models showed an average similarity of $\boldsymbol{BC=0.66}$. Supplementary Figure S2 shows how this cross-validation similarity varies across folds ($\boldsymbol{BC}$ between each fold and the 249 others), and across events. Cross-validation similarity was consistently high (standard deviation of 0.04), supporting the notion that our results are robust and generalisable.

| 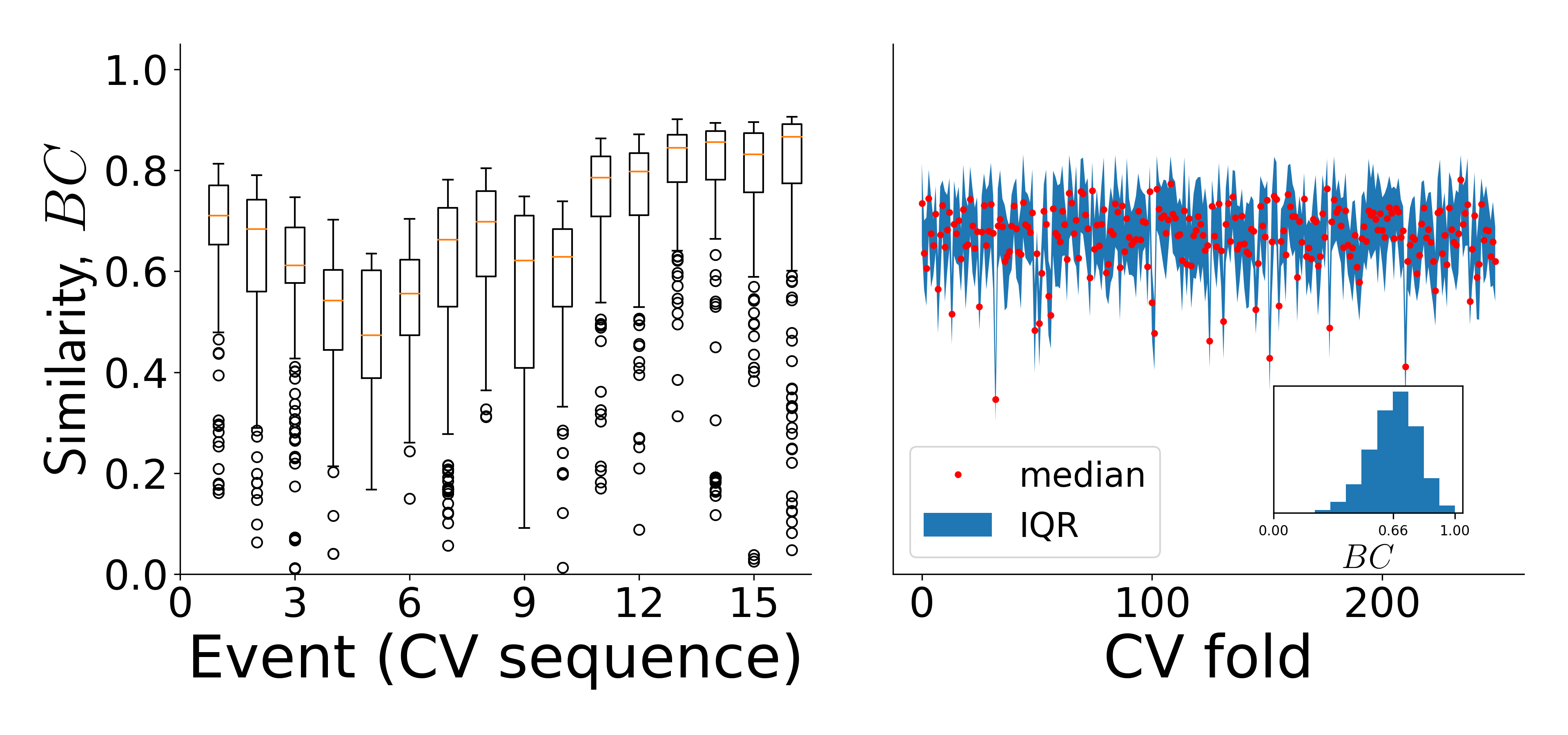 |
| --- |
| Figure S2. Cross-validation similarity across events (left panel, box plots) and folds (right panel, median). High statistical overlap was observed, with an average CV similarity of $\boldsymbol{BC=0.66}$, suggesting a robust sequence of cognitive decline. Abbreviations: CV – cross-validation; BC – Bhattacharyya coefficient. |

## 2.2 Model accuracy: out-of-sample predictions

## For each of the 250 models trained during cross-validation, participants in the test set are staged to assess out-of-sample predictive performance against a ground truth defined as the final cross-validated model stage. This necessitates performing the accuracy calculation *post hoc*, or running a second round of CV. We did the latter.

## Supplementary Figure S3 shows model staging accuracy during cross-validation. Accuracy was consistently high. Mean absolute error within folds varied between 0 and 3 model stages, with an overall mean absolute error of 1.20. This provides further support for the robustness and generalisability of our results.

| 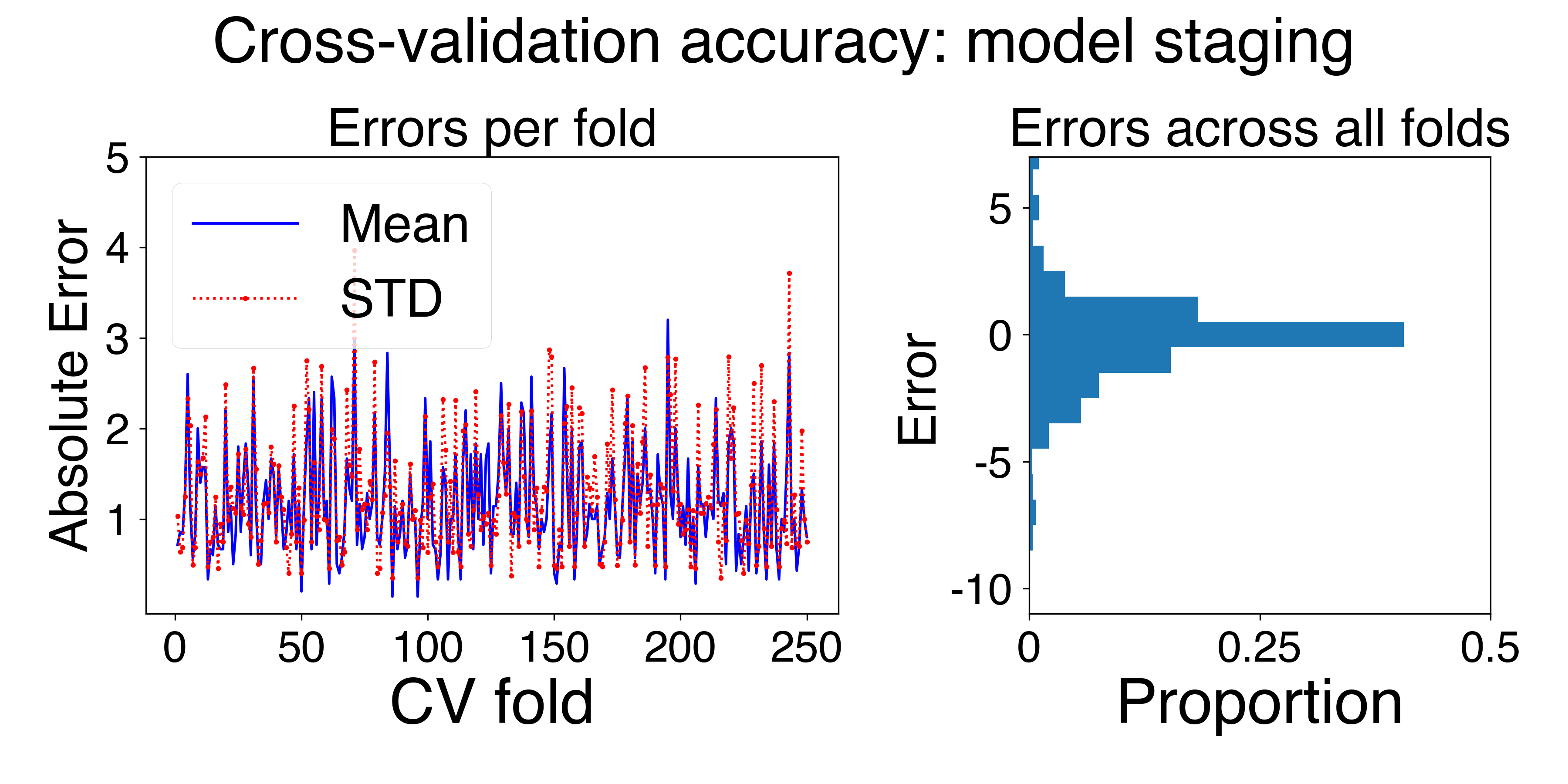 |
| --- |
| Figure S3. Cross-validation accuracy through model staging. Cross-validation accuracy was consistently high across folds, with the mean absolute error consistently below 3 stages. Abbreviations: CV – cross-validation; STD – standard deviation. |

## 2.3 Additional validation: bootstrapping

Repeated stratified k-fold cross-validation is a standard cross-validation strategy for estimating model robustness and out-of-sample predictive performance in the absence of external data for validation. We performed further cross-validation experiments using bootstrap resampling (with replacement). The possibility of selecting individual participants multiple times in each bootstrap sample biases the bootstrapped models towards those individuals, which ultimately produces a final event-based model with higher variability. This has been used widely for finding an upper bound on uncertainty in event-based models (2-5) and elsewhere.

Due to the modest size of our cohort, we estimated event severity measures using the entire cohort, then estimated the sequence by averaging across 100 bootstrap samples from the cohort. This was necessary to improve identifiability of the event severity mixture modelling.

Figure S4 shows the positional density heat maps for all models, side by side: maximum-likelihood (ML, left), cross-validated (CV, centre), and bootstrapped (BS, right). For comparison, each model is displayed using the CV sequence. Qualitatively, the density remains close to the diagonal in all models. Quantitatively, they show high statistical similarity, with Bhattacharyya coefficients (see Supplementary section 2) tightly distributed around 0.66 (±0.04 standard deviation). We also compared the three sequences using Kendall’s tau rank correlation coefficient which varies between –1 and +1 for completely opposite/identical sequences, respectively. Kendall’s tau doesn’t account for the probabilistic nature of our models and so is an underestimate of rank correlation for event-based models. Nevertheless, we found positive rank correlations of $\tau_{CV-ML}=0.18$, $\tau_{CV-BS}=0.25$, and $\tau_{ML-BS}=0.57$. These results provide further support for the robustness and generalisability of our cross-validated event-based model of the sequence of cognitive decline in FAD.

| 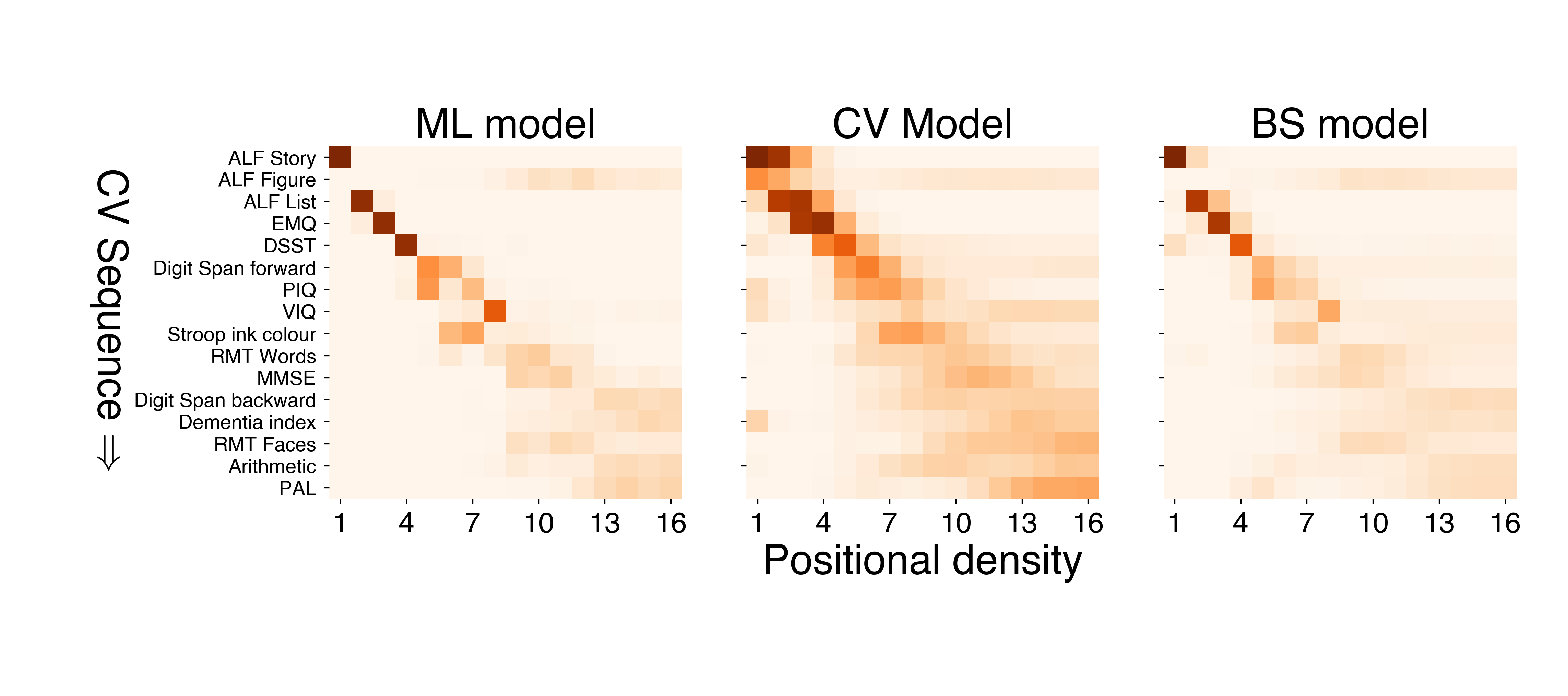 |
| --- |
| **Figure S4.** Validation of the estimated sequence of presymptomatic cognitive decline in FAD from cross-validation (CV sequence, top-to-bottom). Side-by-side comparison of the posterior positional density maps for the maximum-likelihood (ML; left panel), cross-validated (CV; centre panel), and bootstrapped (BS; right panel) models. Qualitative similarity is good, with all three maps showing concentrated density along the diagonal. Quantitative similarity is also high (see text). |

# 3. Methods: event timing

We estimate the timing of cognitive decline by combining the event-based model with EYO — essentially to estimate years between events. We first sum the EBM posterior (positional variance) row-wise to produce cumulative abnormality curves as a function of model stage. We then map model-stage to EYO using linear regression of EYO against model stage in mutation carriers. This produces a group-level, probabilistic estimate of presymptomatic cognitive decline in FAD.

Figure S5 (b) shows the significant linear relationship between EYO and Model stage:
EYO ~ 1.1*(Model stage) – 11.5 (r^2^ = 0.24; p < 0.05). This equates to an average progression rate of approximately 1 model stage per year, starting 11 years before estimated symptom onset.

| 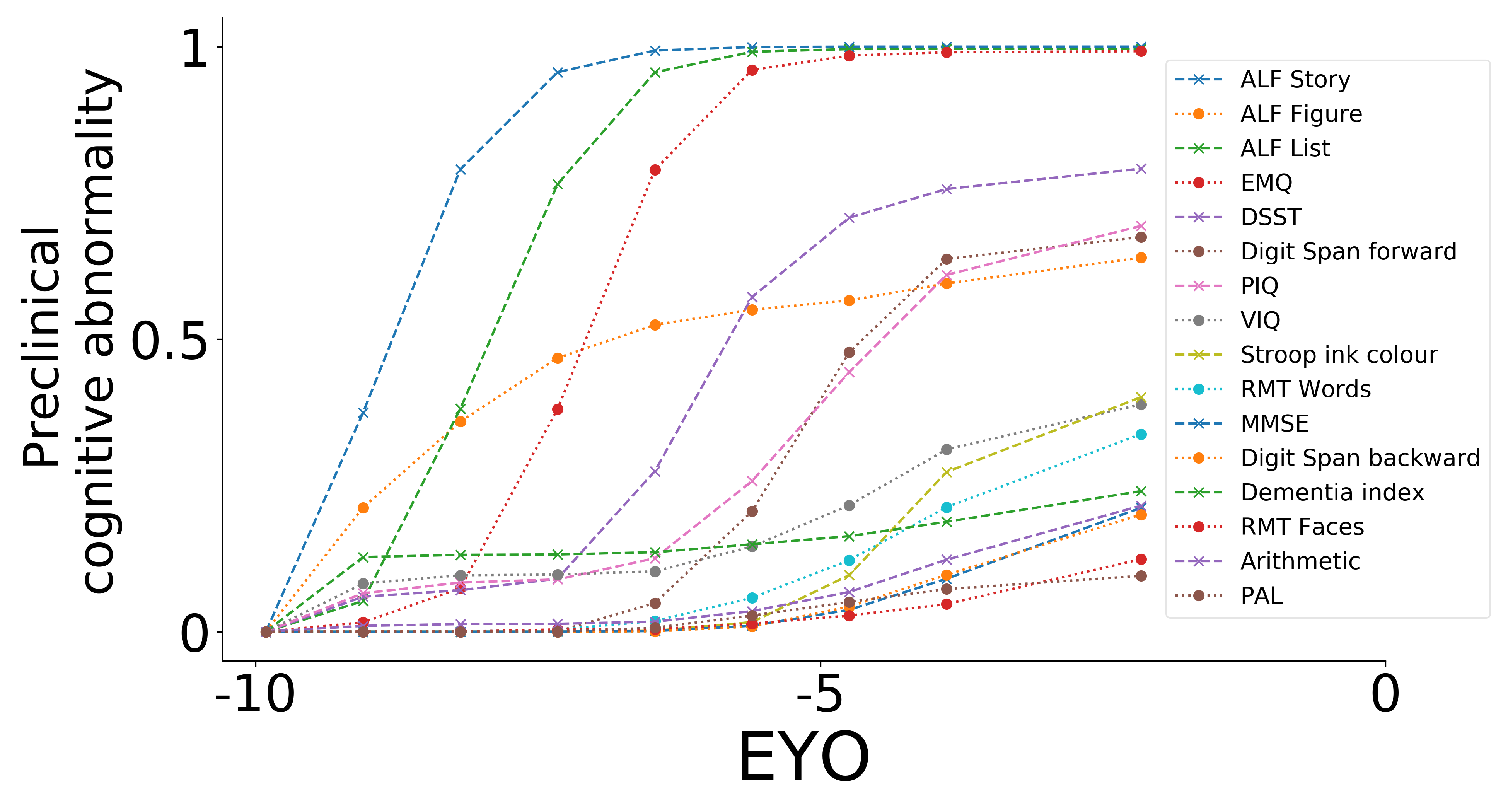 | 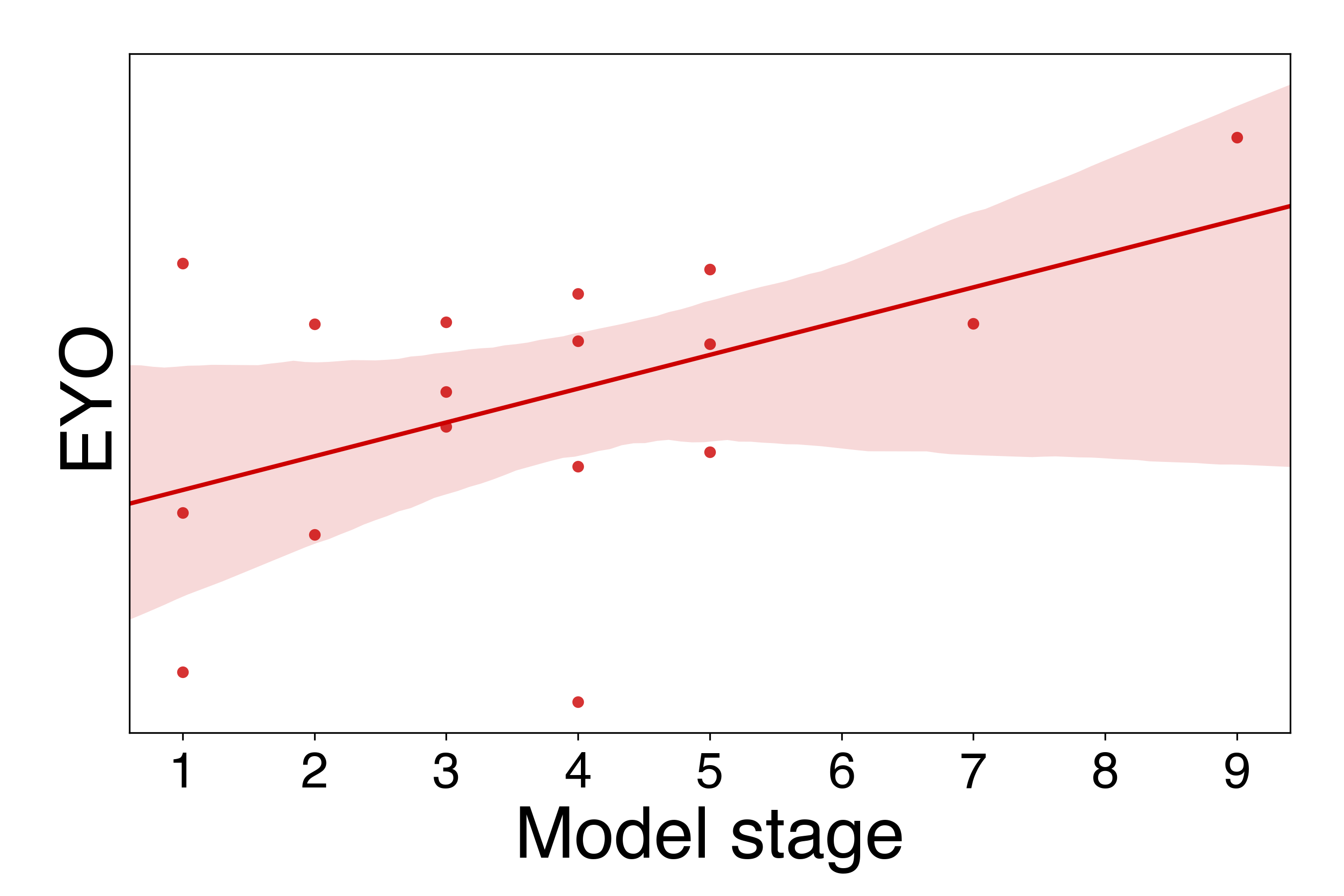 |
| --- | --- |
| 1. Cumulative abnormality from the EBM | 1. Mapping from EBM to EYO |
| **Figure S5.** Timing of events. The cumulative positional density in (a) is mapped to timeline of presymptomatic FAD using (b) linear regression of EYO (unlabelled axis to prevent unblinding) vs model stage in mutation carriers. The resulting curves are shown in the main manuscript (Figure 3). | |

# 4. Cognitive decline with EYO

Figure S6 shows group-level linear regression plots of linear trends for cognitive test scores as a function of EYO, in both mutation carriers (red) and noncarriers (green). Data points and vertical axis labels have been removed to avoid unblinding of research participants and clinicians. Keeping in mind the uncertainty of EYO as a measure of disease progression (3,6), we highlight three key observations. Firstly, it is apparent that tests having strong disease signal (early separation and divergence of trajectories for mutation carriers and noncarriers) appear early in our model: most notably accelerated forgetting (ALF tests) and subjective memory (EMQ). Secondly, it appears that only these first few tests might be useful for distinguishing presymptomatic patients from controls in isolation. This concurs with earlier work on the same cohort (7) and other work on subjective memory complaints (8). This second observation supports the importance of including multiple tests when assessing presymptomatic patients, and the value of using EYO-agnostic approaches such as the event-based model for fusing this information across individuals to improve precision and accuracy for tracking disease progression longitudinally (3). Thirdly, a few tests show apparent (although not statistically significant) practice/learning effects in mutation carriers and not in noncarriers: Verbal IQ, Digit Span forward, MMSE, RMT Faces, and PAL. There are two caveats here: first that these are not true practice effects as the linear regression uses cross-sectional data; secondly, this observation is confounded by EYO inaccuracy but is worthy of further investigation, particularly in larger cohorts using longitudinal data.

| 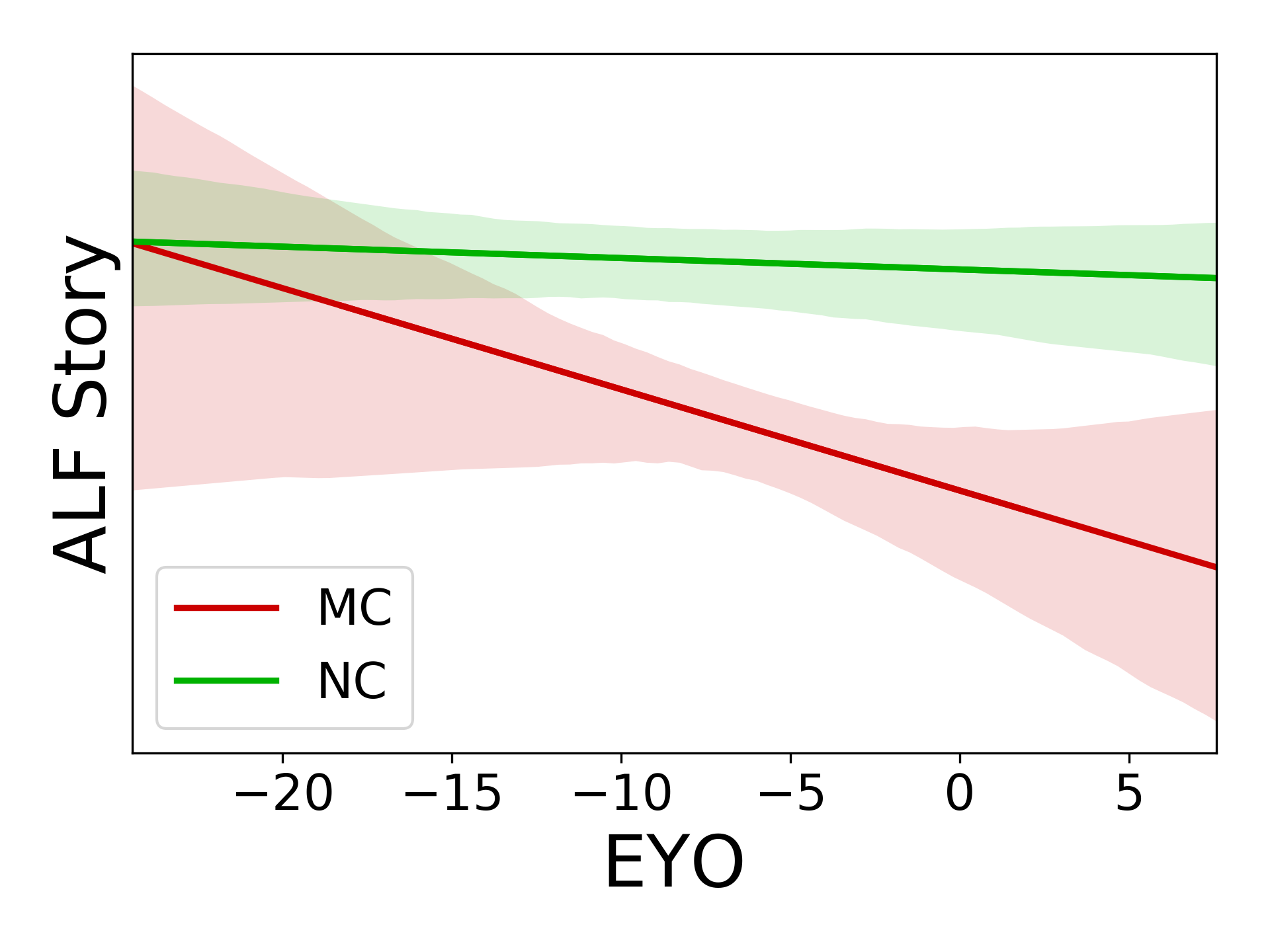 | 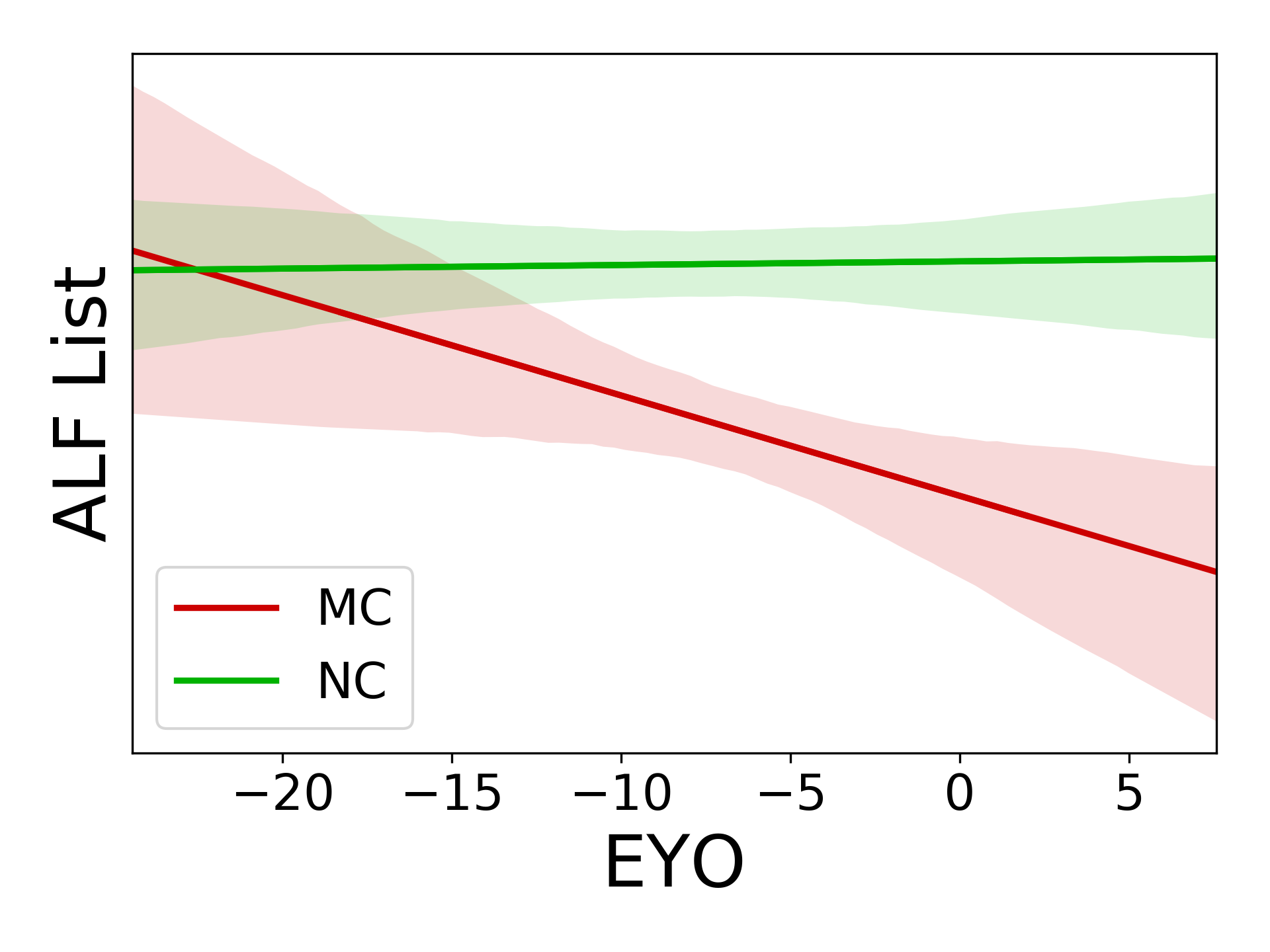 | 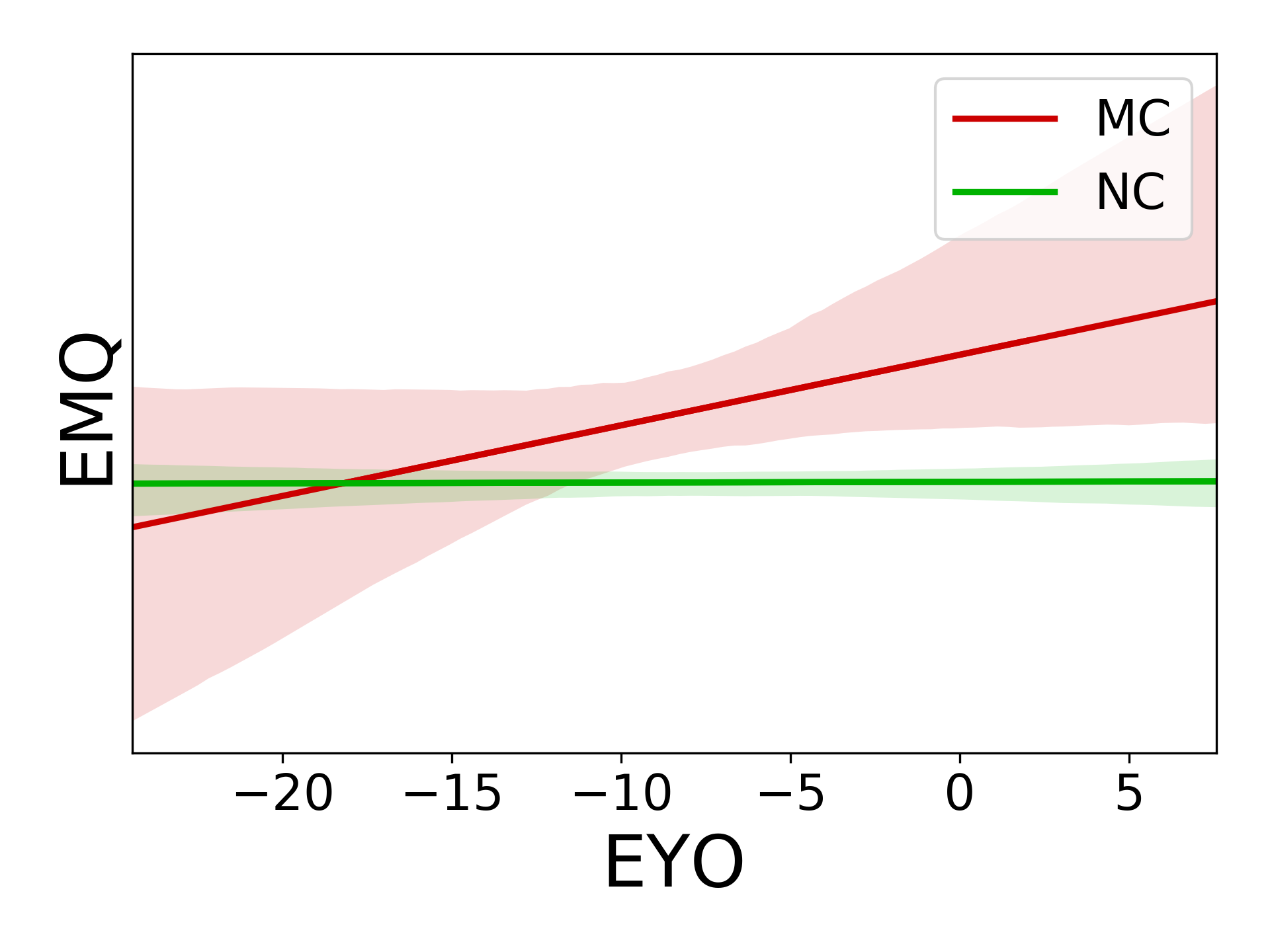 |
| --- | --- | --- |
| ALF Story | ALF List | Everyday Memory Questionnaire |
| 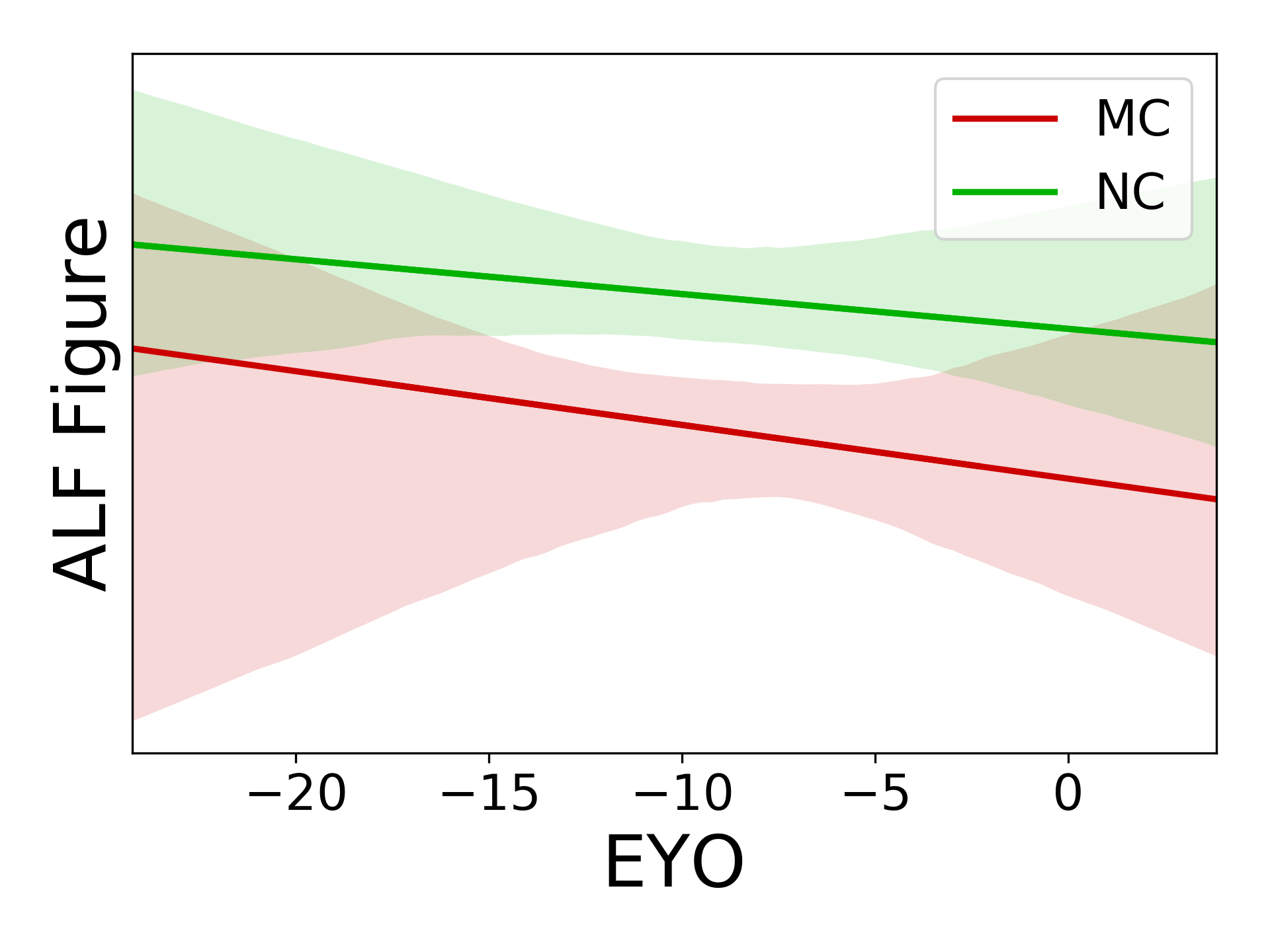 | 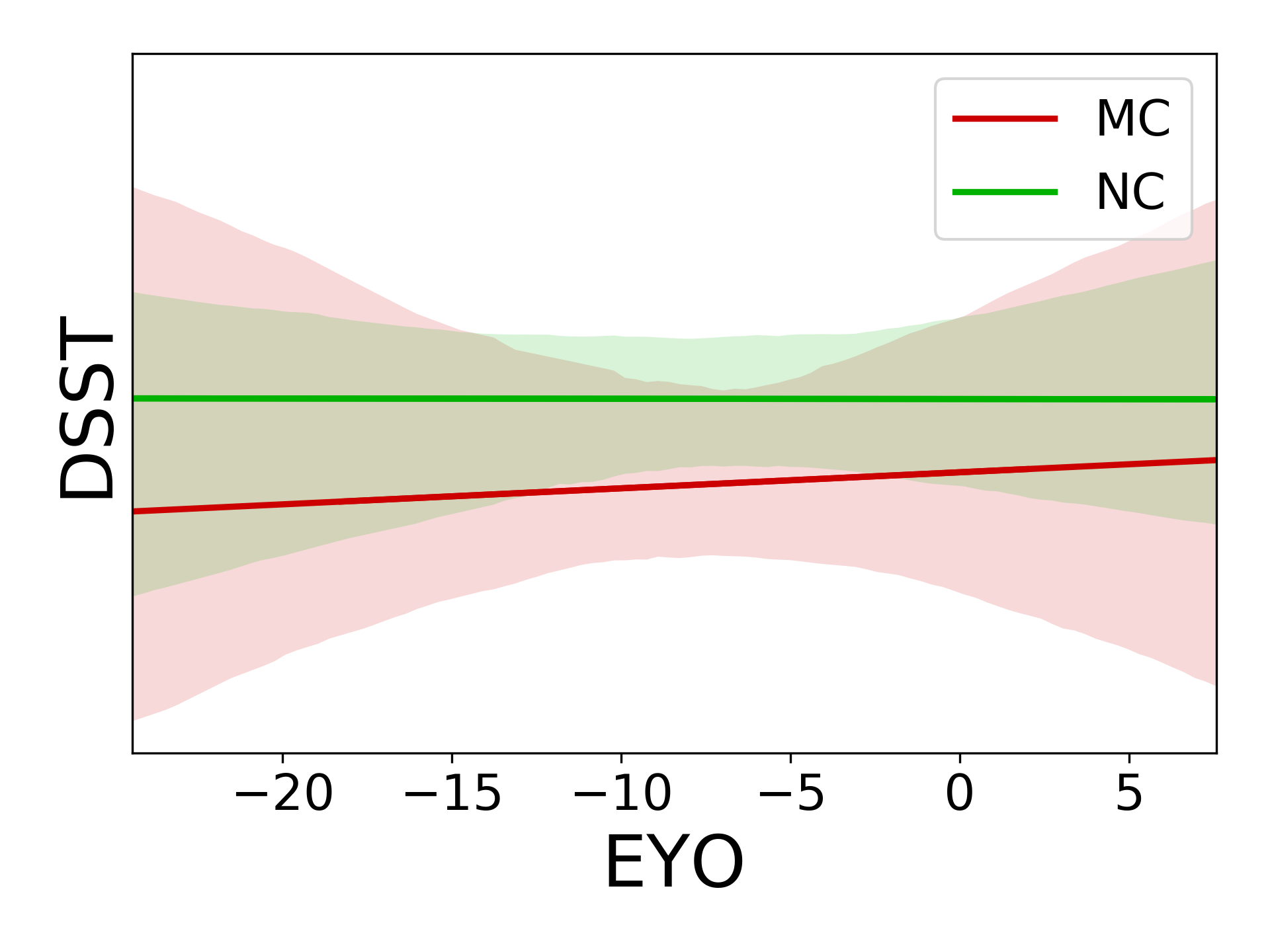 | 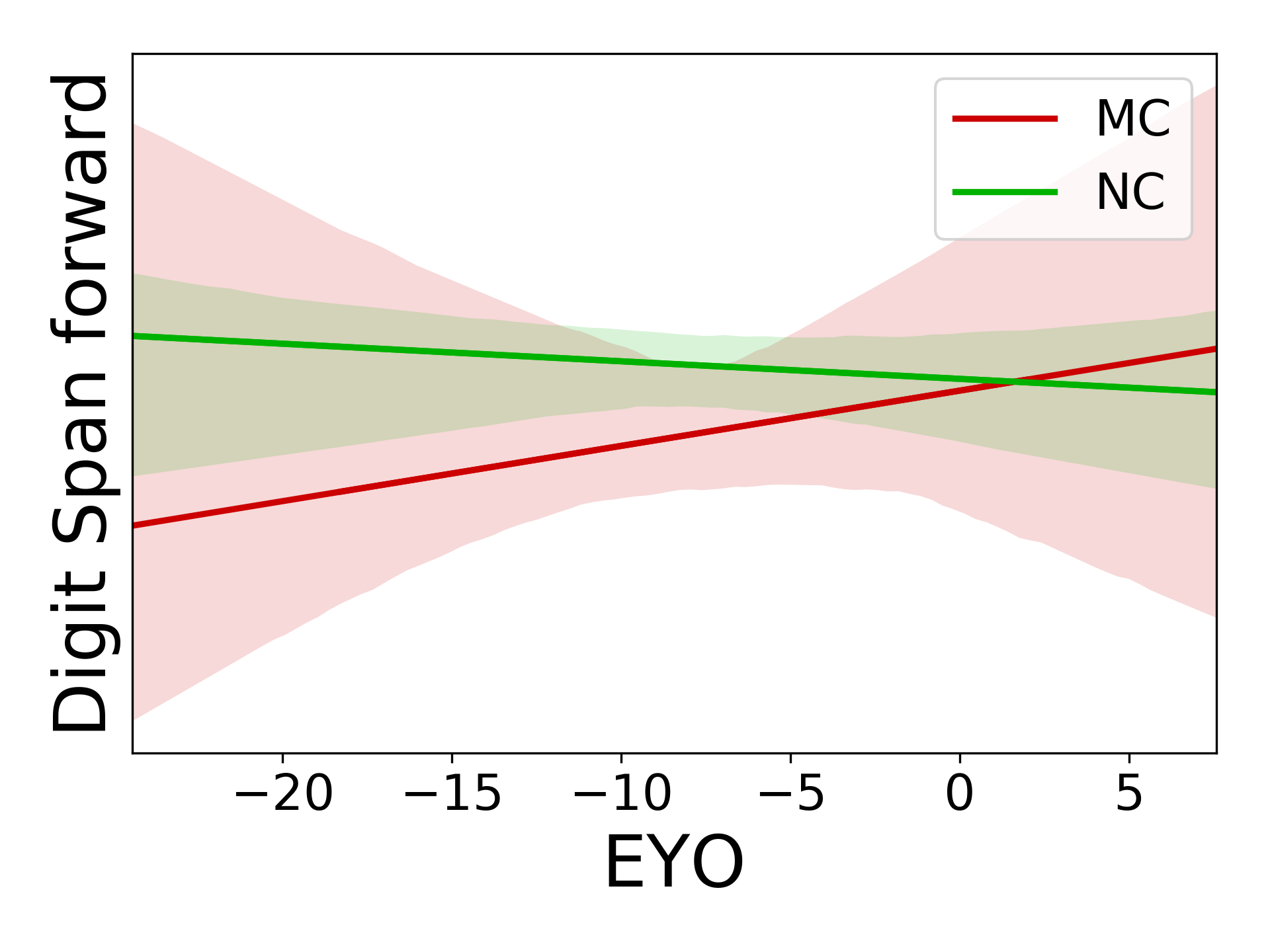 |
| ALF Figure | Digit Symbol Substitution | Digit Span forward |
| 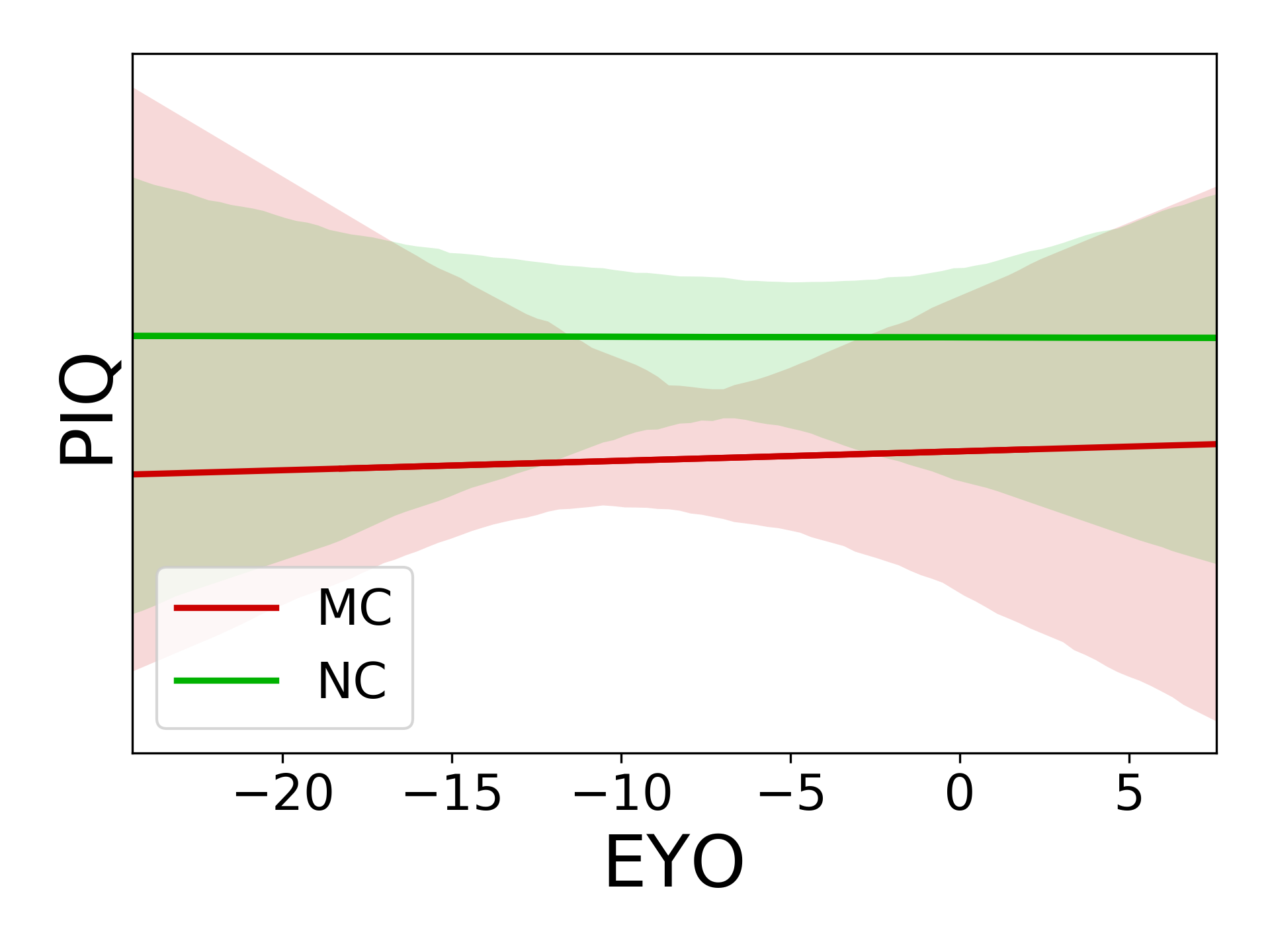 | 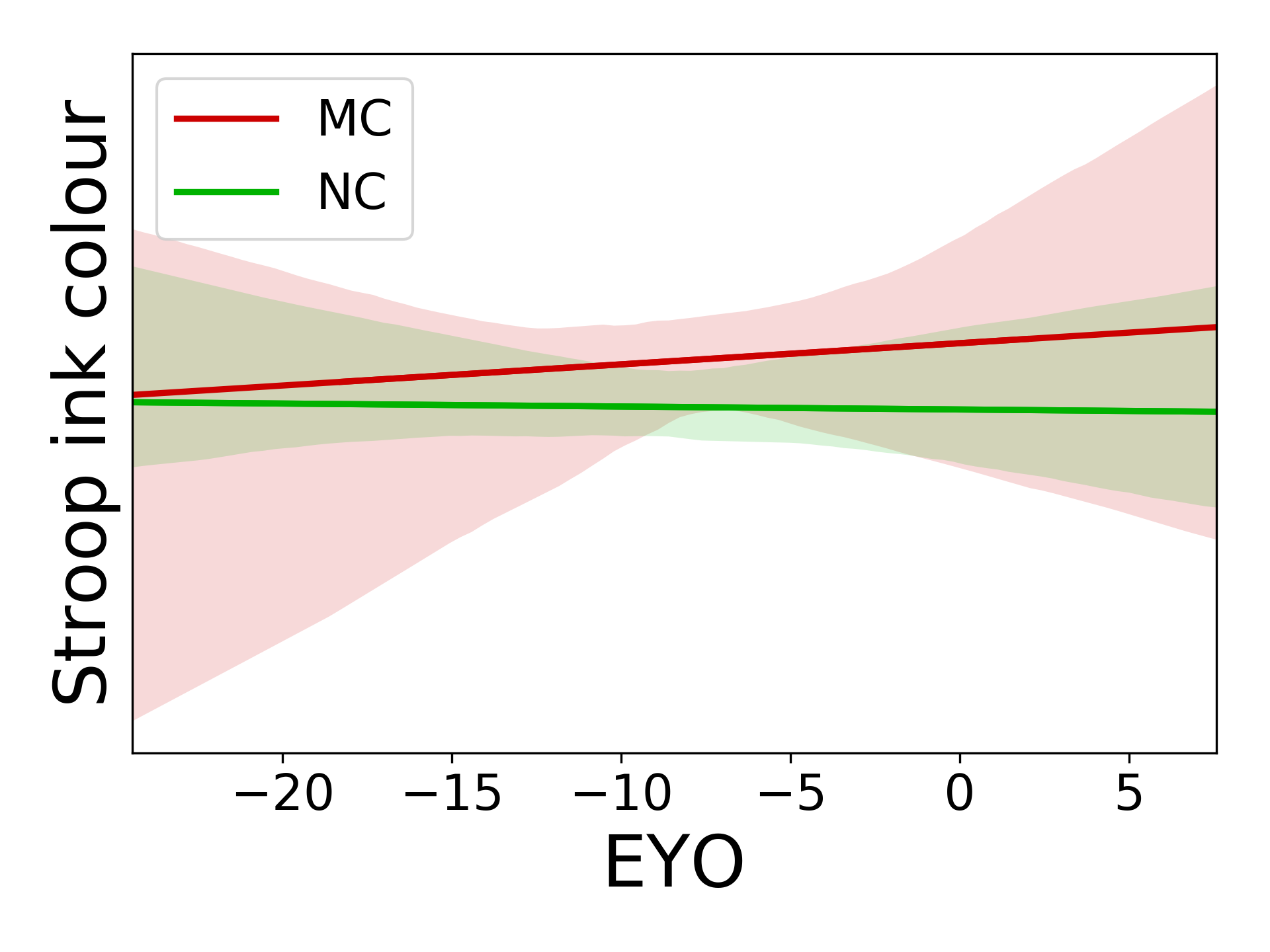 | 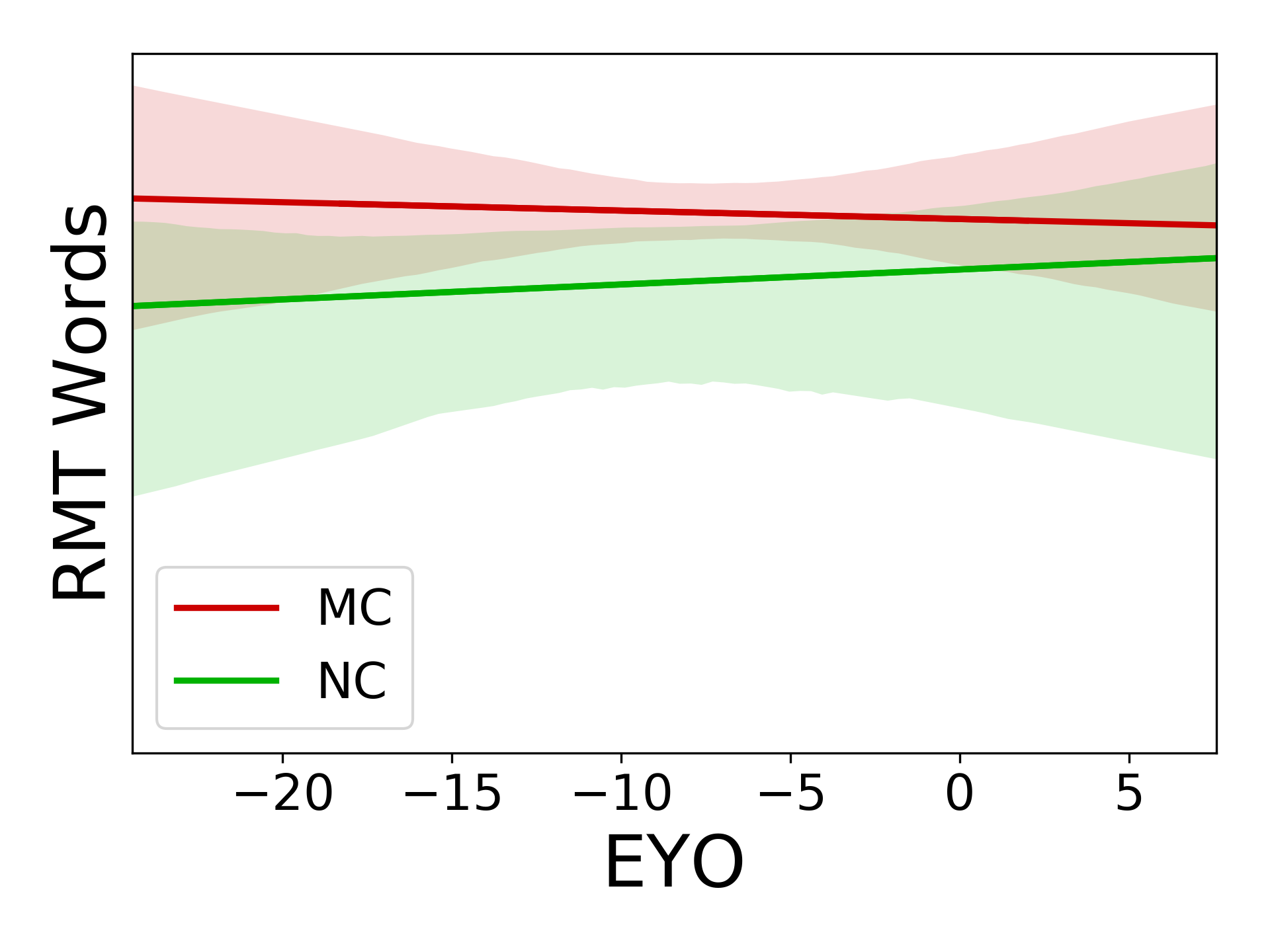 |
| Performance IQ | Stroop ink colour | Recognition Memory: Words |
| 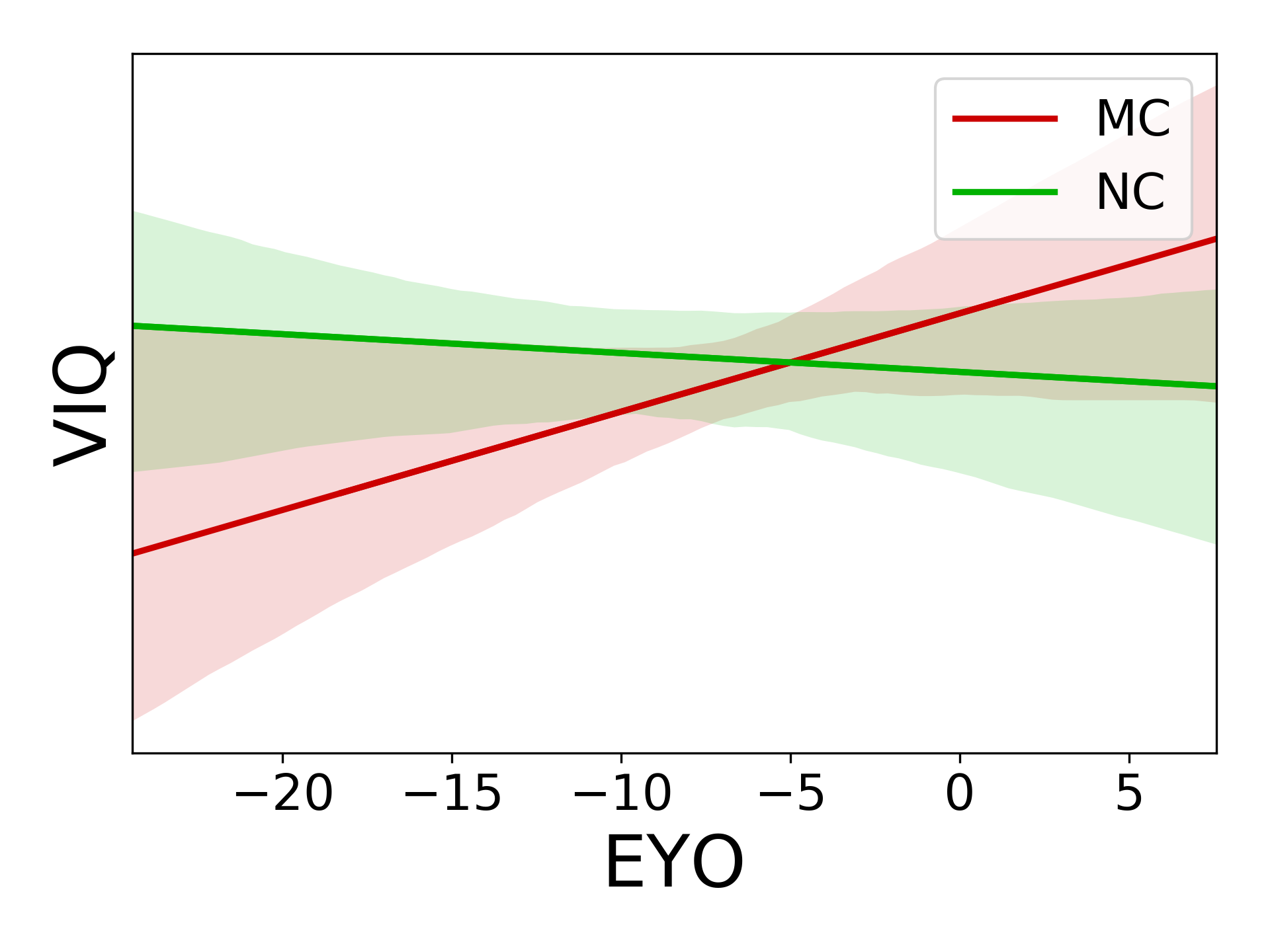 | 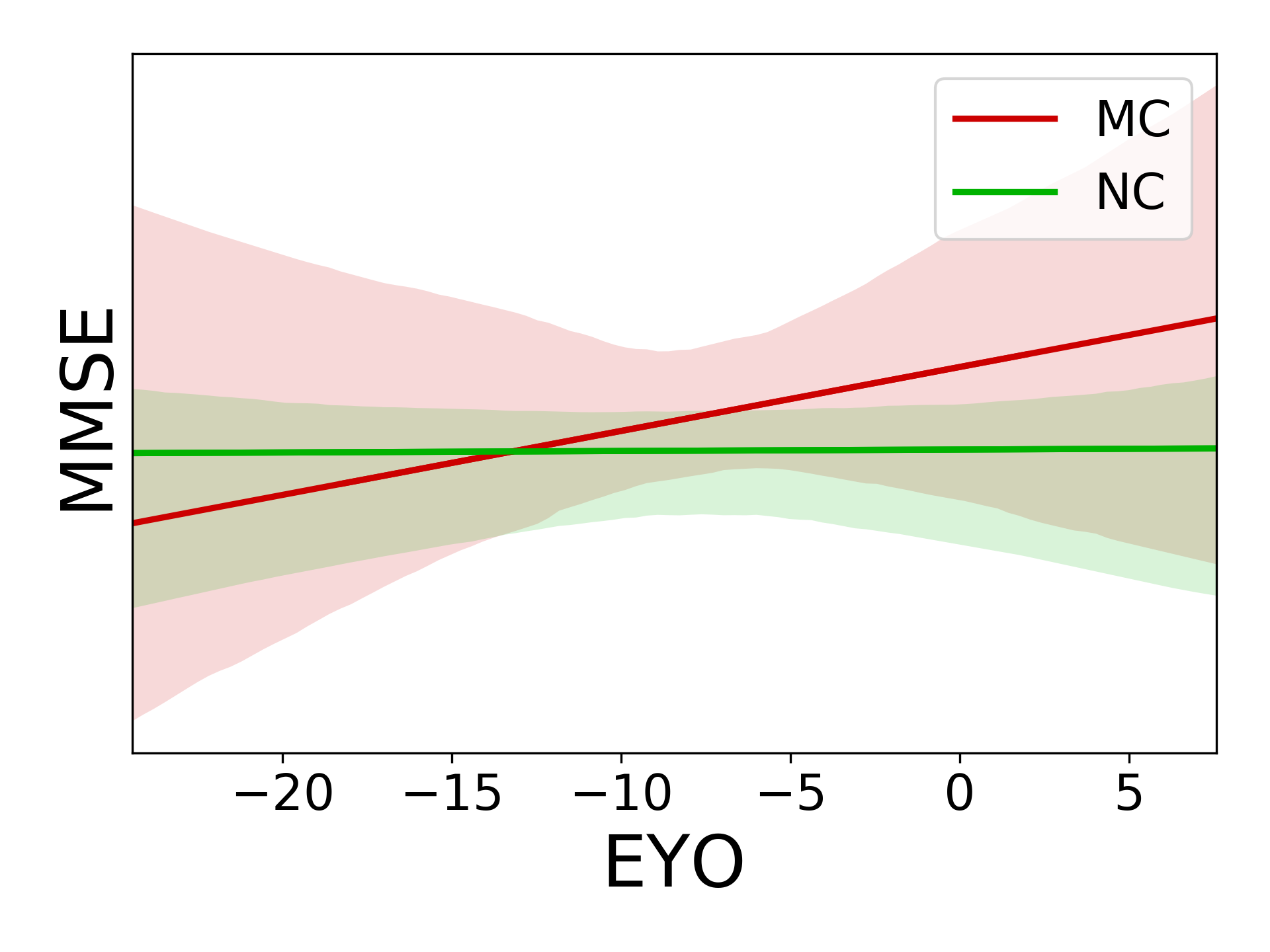 | 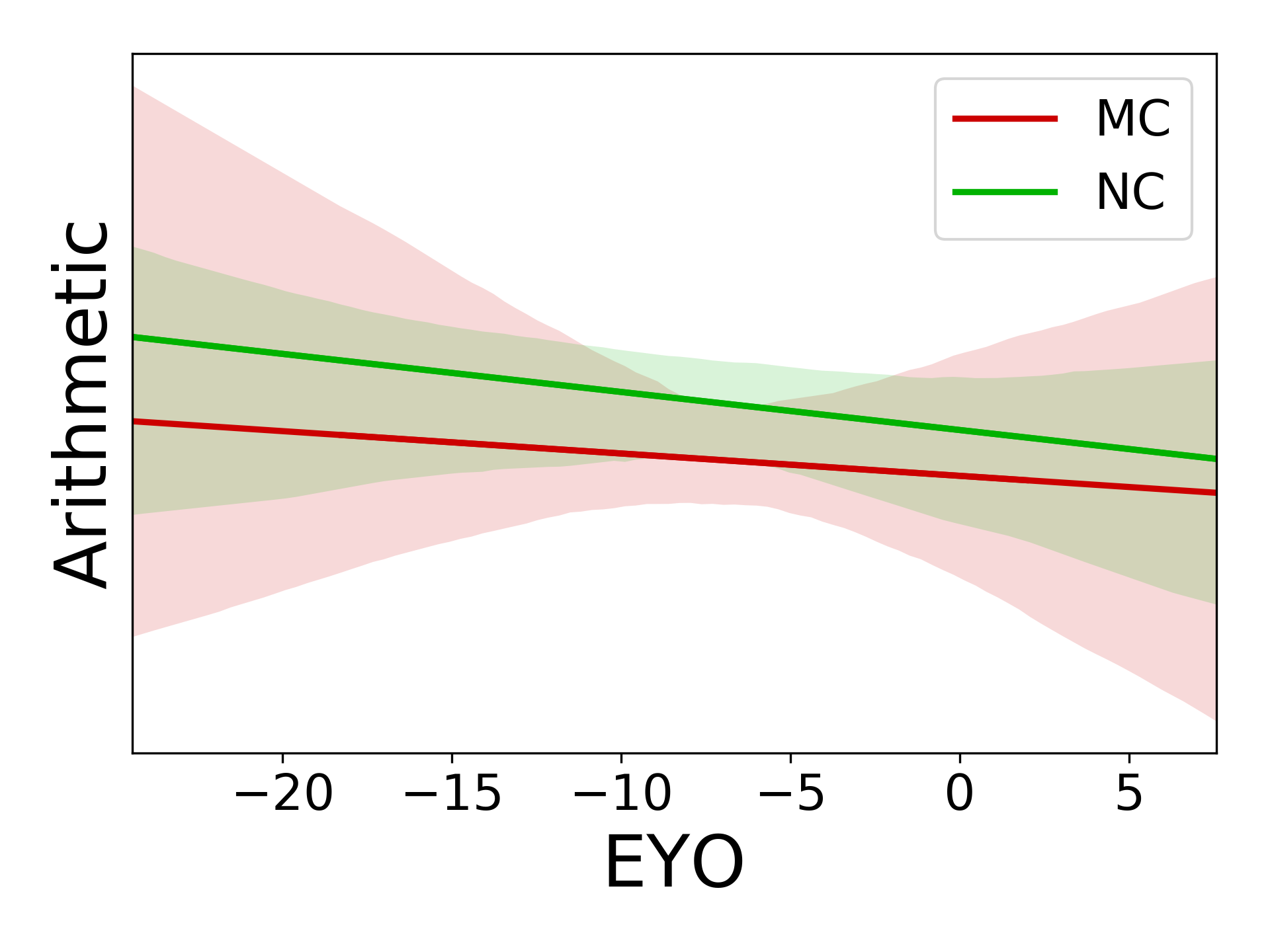 |
| Verbal IQ | MMSE | Graded Difficulty Arithmetic |
| 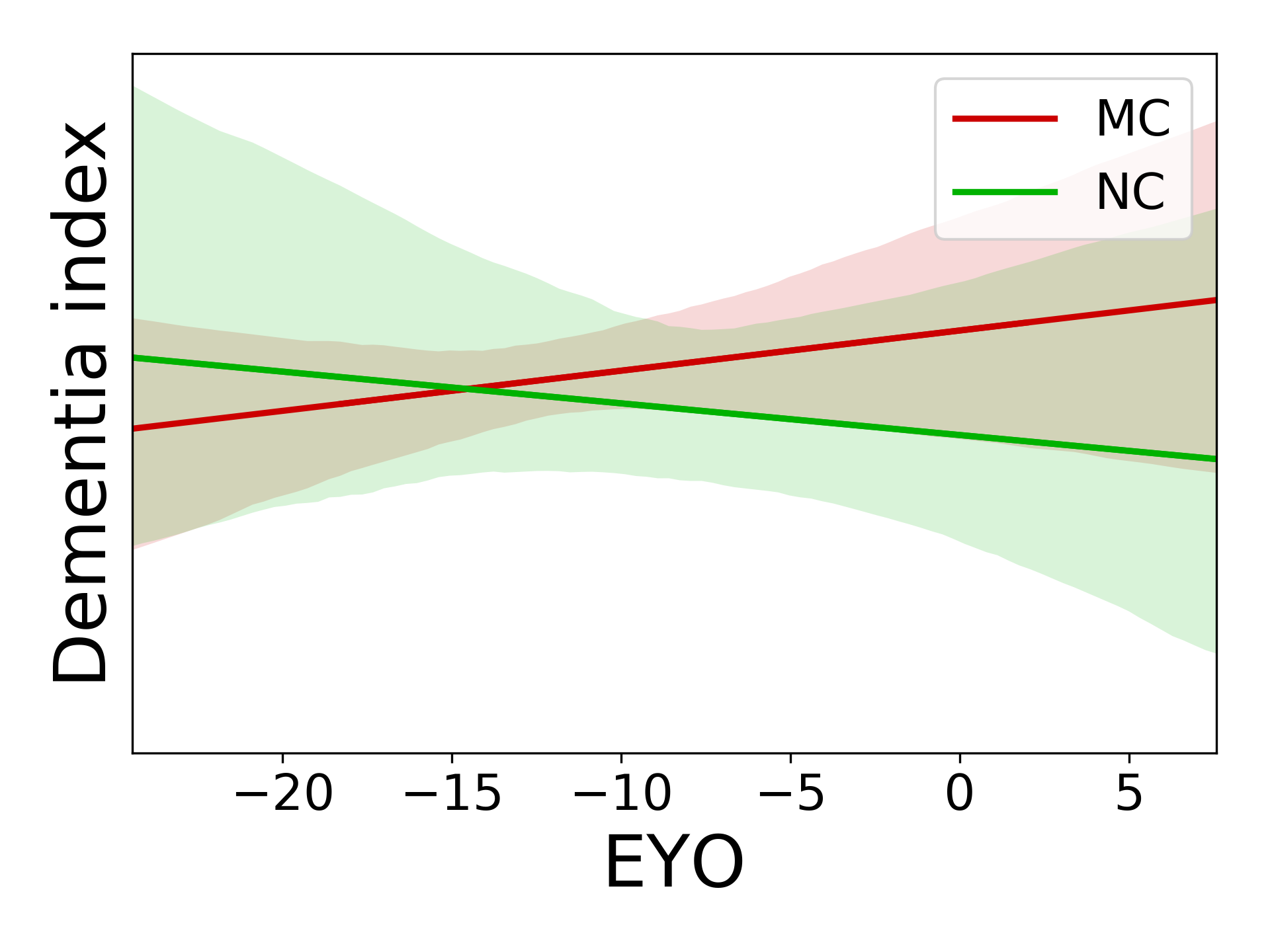 | 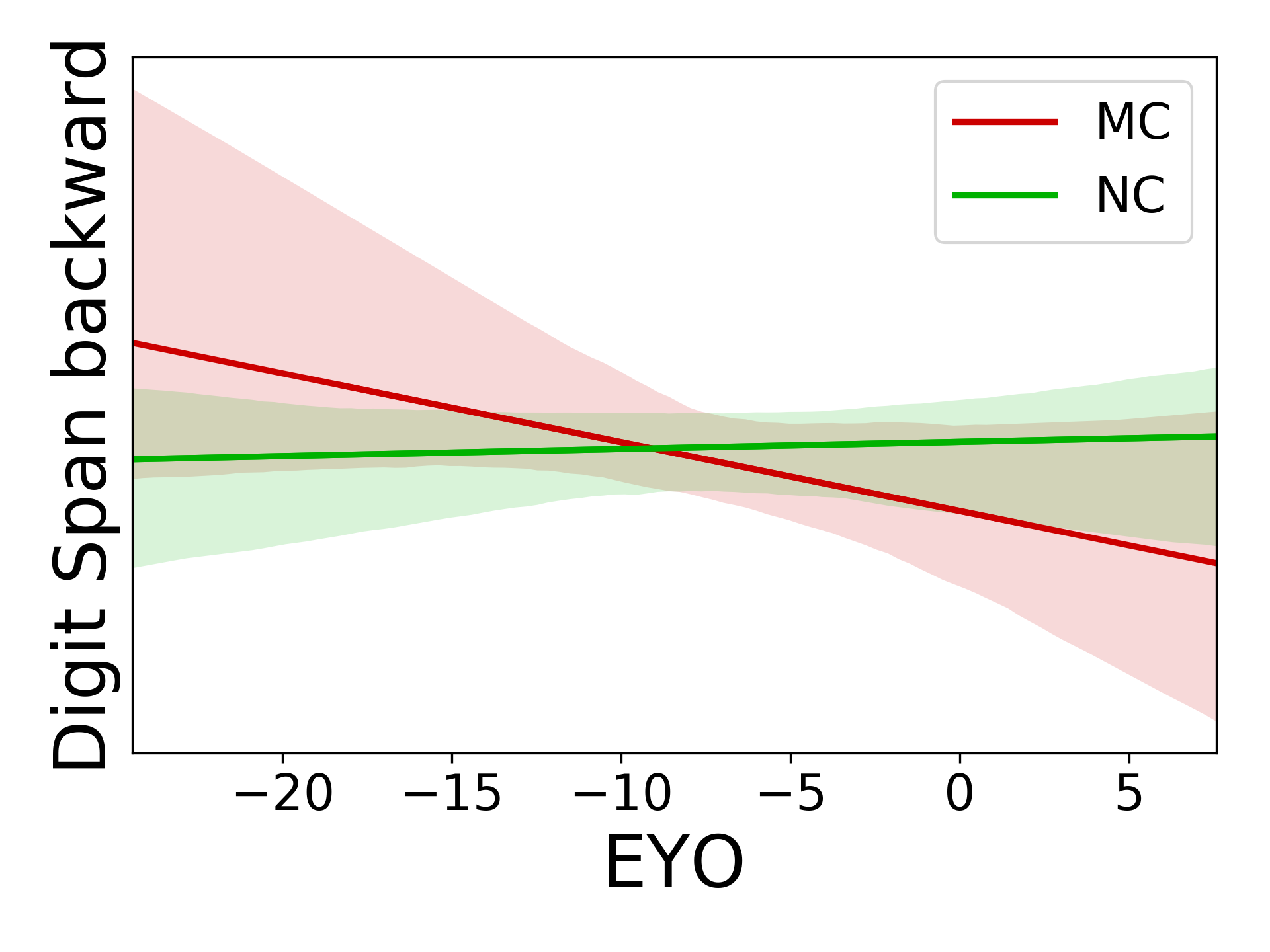 | 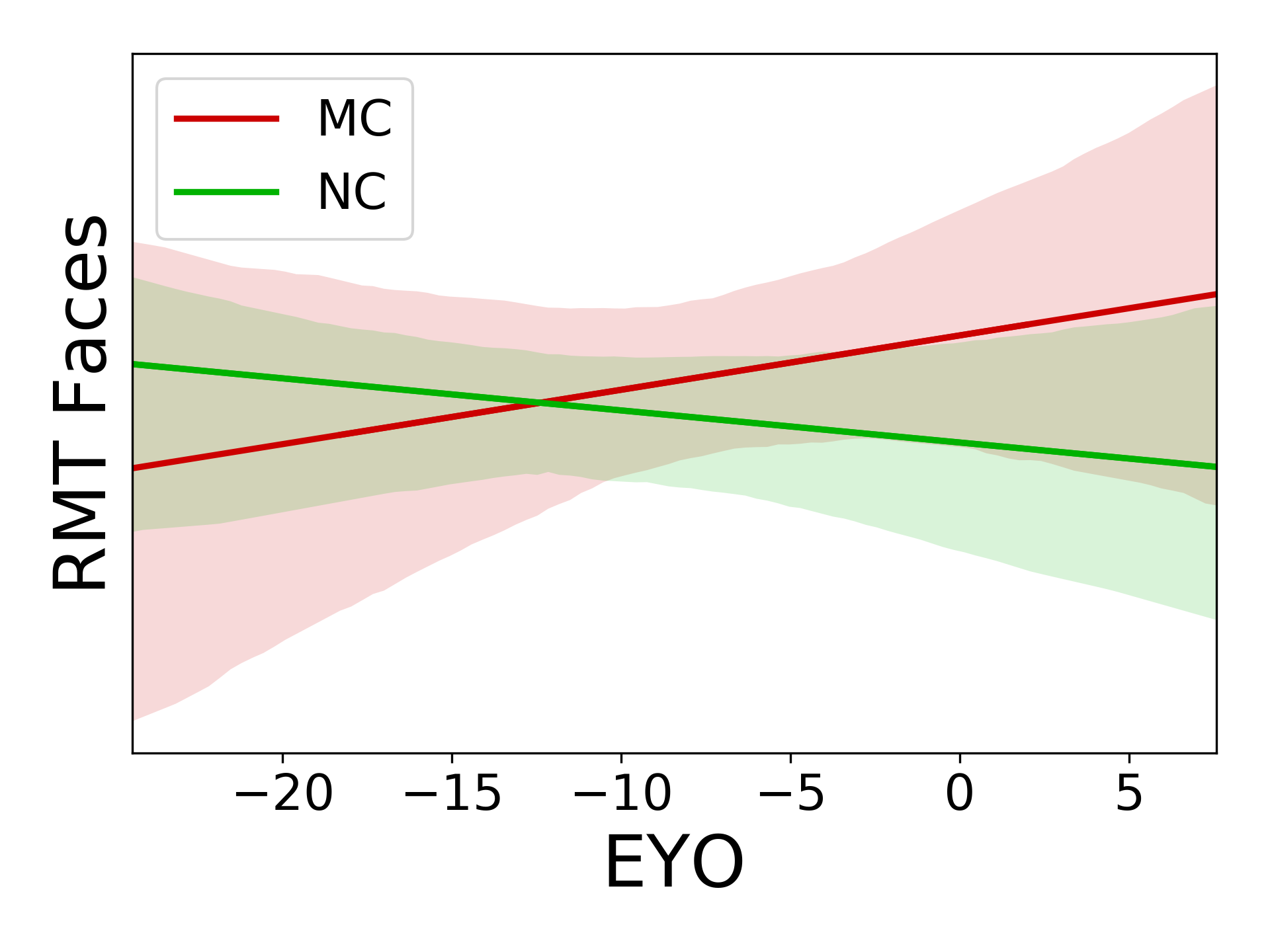 |
| Dementia index | Digit Span backward | Recognition Memory: Faces |
| 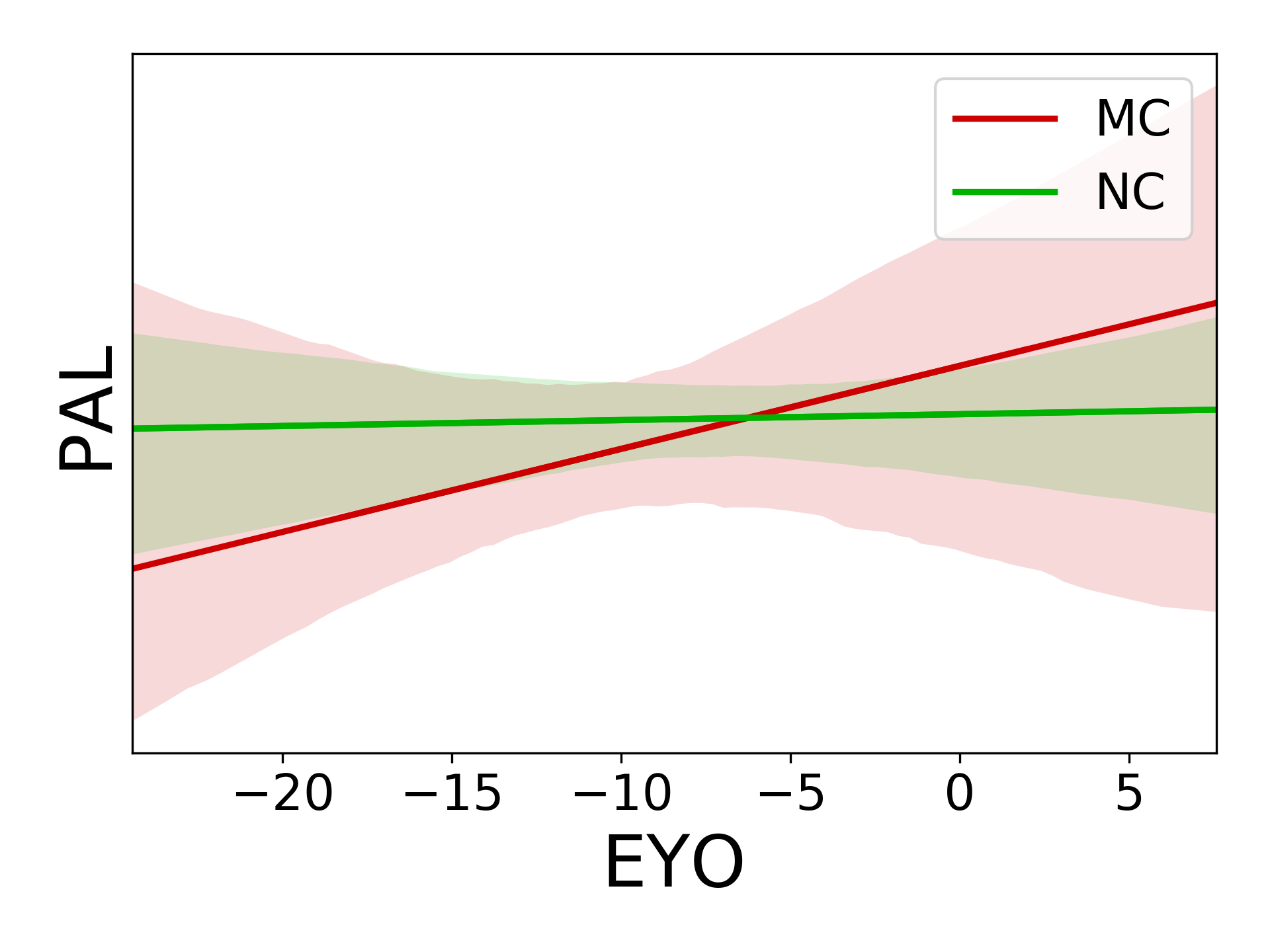 | **Figure S6**. EYO trends for cognitive test scores. Only a few tests show clear separation and divergence between groups in the presymptmotic phase, highlighting the value of using EYO-agnostic methods to assess disease progression in both observational and interventional clinical trials.  Abbreviations: EYO — estimated years to onset. For other abbreviations, see Figure SD. | |
| Paired Associate Learning |  |  |

# 5. Family mutations

| **Gene** | **Mutation** | **Number of individuals** |
| --- | --- | --- |
|  |  |  |
| APP | p.Thr719Asn | 1 AR |
|  | p.Val717Ile | 3 AR |
|  | p.Val717Leu | 1 AR |
| PS1 | Intron 4 | 2 AR |
|  | p.Tyr115His | 1 AR |
|  | p. Tyr115Cys | 1 AR |
|  | p.Ser132Ala | 2 AR |
|  | p.Met139Val | 2 AR |
|  | p.Met146Ile | 3 AR |
|  | p.Glu184Asp | 3 AR |
|  | p.Ile202Phe | 4 AR |
|  | p.His214Tyr | 1 AR |
|  | p.Pro264Leu | 1 AR |
|  | p.Arg278Ile | 3 AR, |
|  | p.Glu280Gly | 6 AR, |
|  | ΔE9* | 1 AR |

**Table e1: The number of individuals from families with each mutation is given.**

Details relating to how many at risk participants for each mutation is given to ensure it is not possible the mutation status of any at risk individual to be revealed/deduced. ** The exon 9 deletion (NM_000021.3:c.869-1G>T; p.Ser290Cys;Thr291_Ser319del) commonly referred to as ΔE9.

**References**

1. Liese F, Miescke KJ. Statistical Decision Theory. In: Statistical Decision Theory: Estimation, Testing, and Selection. New York, NY: Springer New York; 2008. pp. 1–52. (Statistical Decision Theory: Estimation, Testing, and Selection).

2. Young AL, Oxtoby NP, Daga P, Cash DM, Fox NC, Ourselin S, et al. A data-driven model of biomarker changes in sporadic Alzheimer's disease. Brain. 2014 Sep;137(Pt 9):2564–77.

3. Oxtoby NP, Young AL, Cash DM, Benzinger TLS, Fagan AM, Morris JC, et al. Data-driven models of dominantly-inherited Alzheimer’s disease progression. Brain. Oxford University Press; 2018 May 1;141(5):1529–44.

4. Wijeratne PA, Young AL, Oxtoby NP, Marinescu RV, Firth NC, Johnson EB, et al. An image-based model of brain volume biomarker changes in Huntington's disease. Ann Clin Transl Neurol. John Wiley and Sons Inc; 2018 Apr 2;5(5):570–82.

5. Archetti D, Ingala S, Venkatraghavan V, Wottschel V, Young AL, Bellio M, et al. Multi-study validation of data-driven disease progression models to characterize evolution of biomarkers in Alzheimer's disease. NeuroImage: Clinical. 2019;:101954.

6. Ryman DC, Acosta-Baena N, Aisen PS, Bird T, Danek A, Fox NC, et al. Symptom onset in autosomal dominant Alzheimer disease. Neurology. Lippincott Williams & Wilkins; 2014 Jul 15;83(3):253–60.

7. Weston PSJ, Nicholas JM, Henley SMD, Liang Y, Macpherson K, Donnachie E, et al. Accelerated long-term forgetting in presymptomatic autosomal dominant Alzheimer's disease: a cross-sectional study. The Lancet Neurology. Elsevier; 2018 Feb;17(2):123–32.

8. Norton DJ, Amariglio R, Protas H, Chen K, Aguirre-Acevedo DC, Pulsifer B, et al. Subjective memory complaints in preclinical autosomal dominant Alzheimer disease. Neurology. 2017 Oct 3;89(14):1464–70.
